# Alpha–lipoic acid supplementation improves pathological alterations in cellular models of Friedreich ataxia

**DOI:** 10.1186/s13023-025-03990-z

**Published:** 2025-08-23

**Authors:** Marta Talaverón-Rey, Diana Reche-López, Suleva Povea-Cabello, Mónica Álvarez-Córdoba, David Gómez-Fernández, Ana Romero-González, Paula Cilleros-Holgado, José Manuel Romero-Domínguez, Alejandra López-Cabrera, Rocío Piñero-Pérez, Susana González-Granero, José Manuel García-Verdugo, José A. Sánchez-Alcázar

**Affiliations:** 1 Centro Andaluz de Biología del Desarrollo (CSIC-Junta de Andalucía-UPO), Universidad Pablo de Olavide, 41013 Seville, Spain; 2 Laboratory of Comparative Neurobiology, Cavanilles Institute of Biodiversity and Evolutionary Biology, University of Valencia and CIBERNED-ISCIII, Valencia, Spain; 3 Present Address: Department of Biomedical, Sciences, University of Padova, 35131 Padua, Italy

**Keywords:** Friedreich ataxia, Frataxin, Mitochondria, Iron accumulation, Fibroblasts, Induced neurons, α-lipoic acid

## Abstract

**Background:**

Friedreich ataxia (FRDA), the most common autosomal recessive ataxia, is characterized by degeneration of the large sensory neurons and spinocerebellar tracts, cardiomyopathy, and an increased incidence of diabetes. The underlying pathophysiological mechanism of FRDA, driven by a significantly decreased expression of frataxin (FXN), involves increased oxidative stress, reduced activity of enzymes containing iron‑sulfur clusters, defective energy production, calcium dyshomeostasis, and impaired mitochondrial biogenesis, leading to mitochondrial dysfunction.

**Methods:**

This study is aimed at evaluating the role of alpha-lipoic acid (ALA) in reversing the pathological alterations in fibroblasts and induced neurons derived from FRDA patients. Iron accumulation, lipid peroxidation, transcript and protein expression levels of frataxin, mitochondrial proteins, as well as mitochondrial bioenergetics were examined.

**Results:**

Treatment with ALA was able to correct partially the pathological alterations in mutant fibroblasts. The optimal ALA concentration was dependent on the number of expanded GAA triplet repeats in the *FXN* gene. The positive effect of ALA was also confirmed in induced neurons derived from FRDA mutant fibroblasts**.** Our results also suggest that the positive effect of ALA was mediated by Peroxisome Proliferator-Activated Receptor Gamma activation.

**Conclusions:**

Our results suggest that ALA treatment can increase the expression levels of frataxin and reverse the mutant phenotype in cellular models of FRDA.

**Supplementary Information:**

The online version contains supplementary material available at 10.1186/s13023-025-03990-z.

## Background

Friedreich ataxia (FRDA; OMIM #229,300) is an autosomal recessive neurodegenerative disease with a prevalence of approximately 1 in 50,000 individuals, characterized by progressive gait and limb ataxia, sensory loss, muscle weakness, dysarthria, and hypertrophic cardiomyopathy. The disease is caused by pathogenic variants in the *frataxin* (*FXN*) gene that commonly consist of expanded GAA triplet repeats in its first intron resulting in transcriptional silencing and subsequent reduction in frataxin protein expression [1]. While healthy individuals carry fewer than 33 repeats, 96% of FRDA patients are homozygous for a hyperexpansion of GAA codon that can exceed 1000 GAA copies [2, 3]. The remaining 4% of patients are compound heterozygous, possessing a point mutation in one FXN allele and an expanded GAA repeat in the other [4]. The length of the GAA expansion is negatively correlated with the expression of the encoded protein, frataxin [5].

Frataxin is a ubiquitously expressed mitochondrial protein essential for iron homeostasis, biosynthesis of iron-sulfur clusters (ISCs), and cellular energy production [6–9]. Reduction in frataxin expression impairs the activity of ISC-dependent enzymes and the mitochondrial respiratory chain complexes [10], disrupts calcium homeostasis [11–13], promotes mitochondrial iron accumulation [14], increases oxidative stress [15], and results in cell death [16]. These functions affect to the activity of key enzymes such as aconitase and mitochondrial complex I, as well as DNA synthesis and repair mechanisms [17–19]. The primary affected areas include large sensory neurons of the dorsal root ganglia (DRG), the dentate nucleus of the cerebellum, and a variety of other central nervous system (CNS) cells [20–23]. However, cardiomyopathy is the most prominent cause of death for FRDA patients [24].

Depending on the frataxin expression levels, FRDA patients exhibit two different phenotypes: the classical phenotype, characterized by early onset and rapid progression, and the atypical phenotype with late onset and slower progression [25]. This disease is commonly associated with progressive ataxia and muscle dystrophy. In addition, FRDA often leads to severe symptoms, including cardiomyopathy, scoliosis, diabetes, sensorineural hearing loss, and optic neuropathy.

Several experimental models of FRDA have been developed to better understand the disease’s underlying mechanisms, such as *Saccharomyces cerevisiae yhfΔ, Droshophila melanogaster UAS-DfhIR*, and *FXN knock-in* mouse models [26–31]. All these models showed iron accumulation, mitochondrial dysfunction, and high levels of oxidative stress. Nevertheless, the sequence of pathophysiological events underlying FRDA remains poorly understood.

Nowadays, two main strategies are being explored to develop potential treatments for FRDA. The first focuses on increasing frataxin expression levels using genetic therapies, while the second aims to reduce oxidative stress using iron chelators or antioxidants [32].

Due to its antioxidant power and neuroprotective properties, ALA is a promising compound for reducing pathophysiological alterations in cellular models of FRDA. Its effects are attributed to its ability to scavenge reactive oxygen species (ROS), induce the expression of antioxidant systems, and reduce cellular damage. Furthermore, ALA is able to regenerate endogenous antioxidants such as glutathione and vitamin E. Additionally, ALA activates transcription factors, including PPARγ [33–35], Nrf2 or PGC1α, promoting mitochondrial biogenesis [34, 36, 37]. Moreover, it has been demonstrated that PPARγ agonists rescued phenotypic features in cellular and animal models of FRDA [38].

The protective effect of ALA has been suggested in several neurodegenerative diseases such as Alzheimer’s, Huntington’s, and multiple sclerosis. However, further studies are needed to fully elucidate the therapeutic potential and effectiveness of ALA.

This study evaluates the effects of ALA on the pathophysiological features of FRDA in patient-derived cellular models, aiming to determine its therapeutic potential in alleviating oxidative stress and restoring frataxin expression and mitochondrial function.

## Methods

### Reagents

Sudan Black (199664), ( ±) α-LA (62320), Luperox® DI (tert-Butyl peroxide) (168521), Prussian blue (03899), paraformaldehyde (PFA) (158127), dimethyl sulfoxide (DMSO) (17093) and trypsin were purchased from Sigma Chemical Co. (St. Louis, MO). Phosphate-buffered saline (PBS) (102309) was obtained from iNtRON Biotechnology (Seongnam, Republic of Korea).

Anti-TfR1 (13–6800), anti-iron sulfur cluster assembly scaffold protein (ISCU) (MA5-26595), anti-mitochondrial acyl carrier protein (mtACP) (PA5-30099), anti-GPX4 (MA5-32827), propidium iodide (PI) (11539226), anti-NDUFS1 (PA5-22309), Mitotracker™ Deep Red FM (M22426), anti-mouse Alexa Fluor 488 (MN1000), anti-rabbit Alexa Fluor 455 (437695), 4′,6-diamino-2-phenylindole (DAPI) (D1306), bovine serum albumin (BSA) (BP9702), Hoechst 3342 (62249), and Bodipy® 581/591 C11 (D3861) were purchased from Invitrogen™/Molecular probes (Eugene, OR, USA)/ThermoFisher Scientific (Waltham, MA, USA). Anti-Mt-Ft (ab124889), anti-NFS1 cysteine desulfurase (NFS1) (ab58623), anti-LYR motif-containing protein 4 (LYRM4) (ab253001), anti-frataxin (FXN), anti-Nrf2 (ab62352), anti-PGC1α (ab191838), anti-SIRT1 (ab110304), anti PPARγ (ab178860), anti VDAC (ab14734), anti-SDHB (ab14714),Goat Anti-Rabbit IgG H&L (ab6721), Rabbit Anti-Mouse IgG H&L (ab6728), Rabbit Anti-Goat IgG H&L (ab6741), and Aconitase kit were purchased from Abcam (Cambridge, UK). Anti-iron response protein 1 (IRP1) (sc-166022), anti-ferritin light chain (sc-74513), anti-SOD1, anti-catalase (sc-2711803) and deferiprone (sc-211220) were purchased from Santa Cruz Biotechnology (Dallas, TX, USA). Anti-PhosphoPGC1α (S571) was purchased from R&D Systems (Minneapolis, MN, USA). Anti-actin was acquired from MyBiosource (San Diego, California, USA). Anti-lipoic acid antibodies (437695) were purchased from Merck (Rahway, NJ, USA). Anti-TOMM20 antibodies (H00009804-M01) and a cocktail of protease inhibitors (complete cocktail) were purchased from ThermoFisher Scientific (Waltham, MA, USA). The Immun Star HRP substrate kit was from Bio-Rad Laboratories Inc. (Hercules, CA, USA). The information about the reagents is included in Table 1 in Supplementary Material.

### Ethical statements

Approval of the ethical committee of the Hospital Universitario Virgen Macarena y Virgen de Rocío de Sevilla (Spain) was obtained, according to the principles of the Declaration of Helsinki and all the International Conferences on Harmonization and Good Clinical Practice Guidelines.

### Cell cultures

Two cell lines of fibroblasts derived from patient skin biopsies (P1 and P2) and two age- and sex-matched controls fibroblast cell lines from healthy volunteers (C1 and C2) were grown in Dulbecco’s modified Eagle’s medium (DMEM) (Gibco™, ThermoFisher Scientific, Waltham, MA, USA) supplemented with 10% FBS (Gibco™, ThermoFisher Scientific, Waltham, MA, USA), 100 mg/ml penicillin/streptomycin. Fibroblasts were cultured at 37ºC and 5% CO_2_. Patient 1 (P1) harbours 350/350 GAA repeats and patient 2 (P2) carries 450/560 GAA repeats in the first intron of *FXN*. All experiments were performed using cells with a passage number below 12. Patients and control fibroblasts were treated with ALA for seven days.

### Generation of induced neurons (iNs) from control and FRDA (P1 and P2) fibroblasts by direct reprogramming

Neurons were generated from FRDA and control fibroblasts by direct reprogramming as previously described by Drouin-Ouellet et al. [39–41]. Controls and patients-derived fibroblasts were seeded in µ-Slide 4-Well plates (Ibidi Inc., Martinsried, Germany). The day after, dermal fibroblasts were infected with one-single lentiviral vector containing neural transcription factors (Acsl1 and Brn2) and two shRNA against the REST complex, generated as previously described [42]. Cells were infected with a multiplicity of infection (MOI) of 30. The plasmids were a gift from Dr. Malin Parmar (Developmental and Regenerative Neurobiology, Lund University, Sweden). After 24 h, medium was replaced with fresh fibroblast medium. Fibroblasts medium was replaced with neural differentiation medium after 48 h (NDiff227; Takara-Clontech) as described. Neural differentiation medium was refreshed every 48-72 h. On day 21, neural differentiation medium was also supplemented with ALA. Twenty-seven days post-infection neuronal cells were identified by the expression of Tau. DAPI + and Tau + cells were considered induced neurons. Images were taken at DeltaVision system with an Olympus IX-71 fluorescence microscope with 60 × oil objective and analysed by Fiji-ImageJ software, version 2.9.0.

### Iron metabolism

### Prussian blue staining

Iron accumulation was assessed by Perls’ Prussian blue staining in fibroblasts and neurons [43]. Images were taken by light microscopy AE2000 (Motic, Spain) and an integrated camera Axioxam ERc) with a 20X objective and analyzed by FIJI-ImageJ software, version 2.9.0. Iron content was also measured in cell culture extracts using mass spectrometry (ICP-MS) (Tarohda et al., 2005). ICP-MS was performed with an Agilent 7800 mass spectrometer (Agilent Technologies, Sana Clara, CA, USA). Extracts were obtained by acid digestion with HNO_3_.

### LIP determination

We determined labile iron levels by a Calcein-AM (calcein-acetoxymethyl ester, Invitrogen) assay [44]. Cells were incubated with Calcein-AM, which crosses the membrane and reacts with esterases located in the cytosol, resulting in the formation of calcein. Calcein is a fluorochrome that is not able to permeate through membranes, so it remains trapped in the cytosol. Calcein loses its fluorescence when bound to iron and recovers it upon the release of iron, allowing the estimation of intracellular labile iron levels [44].

First, cells were seeded in 12-well plates. 6 × 10^4^ cells were seeded in each well and incubated at 37 °C for 24 h. At the start of the assay, the cells were washed twice with 1X PBS, then cells were incubated with calcein-AM at 0.25 μM for 30 min at 37ºC in loading medium (HBSS supplemented with 20 mM HEPES pH 7.4). After the incubation with Calcein-AM, two washes with 1X PBS were performed, and measurement medium was added (HBSS supplemented with 20 mM HEPES, 150 mM NaCl, 5 mM glucose, pH 7.4). After adding the measurement medium, the first fluorescence reading (F1) was taken using a POLARstar Omega plate reader spectrophotometer (BMG Labtech, Ortenberg, Germany) (excitation: 485 nm; emission: 535 nm). Finally, an iron chelator, deferiprone, was added to the measurement medium at a final concentration of 500 μM, and a second fluorescence measurement (F2) was taken after 1 h. The ratio F2/F1 normalized to the protein amount was used as a relative indicator of the labile iron content.

### Sudan black staining

Lipofuscin accumulation was determined by Sudan Black B (SBB) staining [45] as previously described [46]. SSB staining quantification was assessed by light microscopy. Autofluorescence was assessed by confocal microscopy using an SP5MP-AOBS (Leica, Mannheim, Germany) with a 20X objective. Images were analysed by Fiji-ImageJ software, version 2.9.0.

### Determination of aconitase activity

Both cytosolic and mitochondrial aconitase activity were assessed following the manufacturer’s instructions from Aconitase Assay kit (Biovision, Abcam).

### Evaluation of susceptibility to ferroptosis

Cellular death by ferroptosis was induced by the supplementation of 5 μM of erastin, a ferroptosis activator. A density of 8000 cells/well were plated on 96-multiwells plates. After 24 h, nuclei were stained with Hoechst 3342 (Invitrogen) for 15 min at 37 °C. Cells were rinsed with PBS1X and incubated with 1 μM propidium iodide (PI) and 5 μM erastin. Images were taken every hour using an automatized microscope CellDiscovererer7 (Zeiss,Oberkochen, Germany) at 5X. Nuclei stained with Hoechst and PI were quantified using Stardist from Fiji-ImageJ and cellular death rate was determined.

### Lipid peroxidation

### Measurements of membrane lipid peroxidation

Lipid peroxidation was evaluated using 4,4-difluoro-5-(4-phenyl- 1,3-butadienyl)-4-bora-3a,4a-diaza-s-indacene-3-undecanoic acid (BODIPY® 581/591 C11), a lipophilic fluorescent dye (Alcocer-Gómez et al., 2015; Pap et al., 1999). Cells were incubated with 5 μM BODIPY® 581/591 C11 for 30 min at 37 °C. 500 μM Luperox® for 15 min were used as positive control of lipid peroxidation. Lipid peroxidation in fibroblasts was evaluated by an Axio Vert A1 fluorescence microscope with a 20X objective. Images were analysed with Fiji-ImageJ software, version 2.9.0.

### Transmission electron microscopy (TEM) analysis

Cells were seeded in 8-well Permanox chamber slides (177,380, ThermoFisher Scien-tific, Waltham, MA, USA) in DMEM glucose medium for three days. Cells were fixed with 3.5% glutaraldehyde in 0.1 M phosphate buffer for 10 min at 37 °C. After fixation, cells were post-fixed in 2% OsO_4_, rinsed, dried, and embedded in Durcupan Resin (44,610-1EA, Sigma-Aldrich (Saint Louis, MO, USA)). Using a diamond knife, ultrathin pieces of 70 nm were cut and examined using a transmission electron microscope (FEI Tecnai G2 Spirit BioTwin) equipped with a Xarosa digital camera (20 Megapixel resolution) and Radius image acquisition software version 2.1 (EMSIS GmbH, Münster, Germany).

### Protein expression levels

### Immunoblotting

Western blotting was performed using standard Methods. After transferring protein to a nitrocellulose membrane, the membrane was incubated with primary antibodies diluted 1:1000, and then with the corresponding secondary antibody coupled to horseradish peroxidase at a 1:2500 dilution. Specific proteins were identified by ChemiDoc™ MP Imaging System (Bio-Rad, Hercules, CA, USA) using the Immun Star HRP substrate kit (Biorad Laboratories Inc., Hercules, CA, USA). ImageLab™ version 10.0 software (Bio-Rad, Hercules, CA, USA) was used to analyze protein expression levels.

#### Immunofluorescence microscopy

iNs were plated on μ-Slide 4-well plates (Ibidi Inc., Martinsried, Germany). Cells were rinsed once with PBS, fixed in 3.8% paraformaldehyde for 10 min at room temperature. Then, cells were permeabilized with 0,1% Triton X-100 for 10 min. Next, cells were incubated with a blocking solution containing 5% donkey serum for 1 h. Primary antibodies, diluted 1:200–1:500 in blocking solution, were incubated overnight at 4 °C. Unbound antibodies were removed by washing twice with PBS. Cells were incubated with secondary antibodies, diluted 1:300 in blocking solution, for 2 h at room temperature. Finally, cells were stained with 1 μg/mL DAPI for 15–20 min. Images were taken with a DeltaVision system with an Olympus IX-71 fluorescence microscope with a 60 × oil objective and analysed by Fiji-ImageJ software, version 2.9.0.

### Frataxin transcript expression levels

### Real-time quantitative PCR (qPCR)

*FXN* gene expression in fibroblasts was analysed by qPCR using mRNA extracts. mRNA was isolated with Trizol™ (Invitrogen, Carlsbad, CA, USA), following manufacturer’s instructions. RNA was retrotranscribed using Iscript cDNA synthesis Kit (Bio-Rad,Hercules, CA, United States) to obtain complementary DNA (cDNA). qPCR was performed using TB Green™ Premix Ex Taq™ (Takara Bio Europe S.A.S., Saint-Germain-en-Laye, France). CFX Connect Real-Time PCR Detection System (Bio-Rad, Hercules, CA, USA) was used to detect accurate quantification of gene expression. Frataxin primers were 5’-CCTTGCAGACAAGCCATACA-3’ (Forward primer) and 3’-CCACTGGATGGAGAAGATAG- 5’ (Reverse primer), amplifying a sequence of 149 nucleotides. Actin was used as a housekeeping control gene and the primers were 5’- AGAGCTACGAGCTGCCTGAC -3’ (Forward primer) and 3’- AGCACTGTGTTGGCGTACAG -5’ (reverse primer).

### Mitochondrial function

### Assessment of mitochondrial respiratory function using Seahorse extracellular flux analyzer

Mitochondrial respiratory function and oxygen consumption rate (OCR) of control and FRDA fibroblasts were measured using Mito-stress test assay in a XFe24 extracellular flux analyzer (Seahorse Bioscience, Billerica, MA, USA). Cells were seeded in XF24 cell culture plates with a density of 10,000 cells/well and placed in a 37 °C incubator with 5% CO_2_.

The day after, cells were washed twice with 500 μL of pre-warmed assay medium (XF base medium supplemented with 10 mM glucose, 1 mM glutamine and 1 mM sodium pyruvate; pH 7.4 Cells were placed in a 37 °C incubator without CO_2_ for 1 h to allow equilibrating with the assay medium. Pre-warmed oligomycin, FCCP and rotenone & antimycin A were loaded into the ports A, B and C of sensor cartridge, respectively. The final concentrations of injections were as follows: 1.5 μM oligomycin, 2 μM FCCP, 1 and 2.5 μM rotenone & antimycin A, respectively.

The cartridge was calibrated by the XFe24 analyzer, and the assay was carried out using Mito-stress test assay protocol. OCR was measured followed by the sequential addition of oligomycin, FCCP and rotenone & antimycin A. This approach allows us to determine different parameters of mitochondrial function such as basal respiration, maximal respiration, spare respiratory capacity and ATP production. When the experiment was finished, cell number of each well was calculated by performing a staining with DAPI using the BioTek™ Cytation™ 1 Cell Imaging Multi-Mode Reader (Biotek, Winooski, VT, USA).

### Statistical analysis

Statistical analysis was performed as formerly described by our research group [47]. We used non-parametric statistics that do not have any distributional assumption in cases when the number of events was small (n < 30) [48]. In these cases, multiple groups were compared using a Kruskal–Wallis test. In cases when number of events was higher (n > 30), we applied parametric tests. In these cases, multiple groups were compared using a one-way ANOVA. Statistical analyses were conducted using the GraphPad Prism 9.0 (GraphPad Software, San Diego, CA). The data are reported as the mean ± SD values or as representative of at least three independent experiments. P-values of less than 0.05 were considered significant.

## Results

### Pathophysiological characterization of FRDA fibroblasts

#### FRDA fibroblasts show low frataxin protein and transcript expression levels

**Fig. 1:**
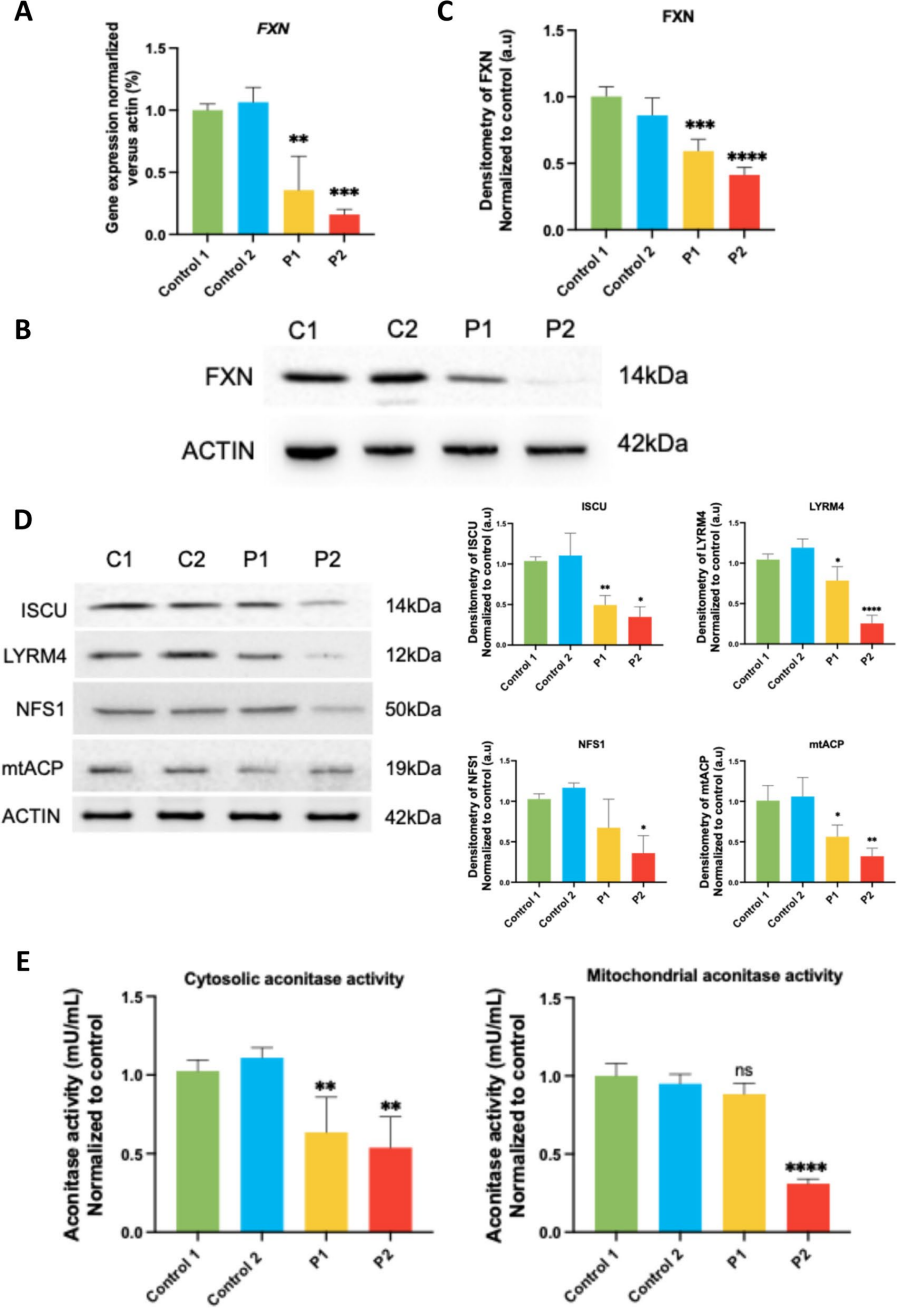
Frataxin expression levels, Fe-S assembly proteins and aconitase activity expression in mutant FXN fibroblasts. **A** FXN transcripts from controls (C1 and C2) and two FRDA fibroblast cell lines (P1, P2) were quantified by RT-qPCR. Data represent the mean ± SD of three separate experiments. **B** Immunoblotting analysis of cellular extracts from controls (C1 and C2) and FRDA patient cell lines (P1 and P2). Protein extracts (15 μg) were separated on a SDS polyacrylamide gel and immunostained with antibodies against FXN. Actin was used as a loading control. **C** Densitometry of FXN. **D** Immunoblotting analysis of cellular extracts from control (C1 and C2) and FRDA fibroblasts, P1 and P2. Protein extracts (15 μg) were separated on a SDS polyacrylamide gel and immunostained with antibodies against ISCU, LYRM4, NFS1 and mtACP. Actin was used as a loading control. **E** Measurement of aconitase activity. Mitochondrial and cytosolic extracts were separated and quantified to determine cytosolic and mitochondrial aconitase activity by colorimetric assay. Data represent the mean ± SD of three separate experiments. Statistical significance between control and FRDA fibroblasts is expressed *p < 0.05, **p < 0.01, ***p < 0.001 ****p < 0.0001

First, we assessed the expression of the *FXN* gene at the transcriptional and protein level. RT-qPCR assays confirmed a decrease in *FXN* gene expression in patient-derived fibroblasts (Fig. 1A). Specifically, P1-derived fibroblasts, which contain 350 GAA repeats in each allele, exhibited a 60% reduction in gene expression, while P2-derived fibroblasts, with 450 and 560 GAA repeats in each allele, showed an 80% reduction in comparison to control fibroblasts. Frataxin protein levels, determined by Western blot analysis, were also significantly reduced in both patient-derived fibroblast lines. Notably, we observed a greater reduction of FXN protein expression levels in P2 fibroblasts (Fig. 1B and C).

#### FRDA fibroblasts manifest impaired ISC protein expression levels

Since frataxin has an important role in Fe-S cluster assembly, we decided to evaluate the expression levels of proteins also involved in this process such as ISCU, NFS1, LYRM4 and mtACP. Western blot assays showed that these proteins were downregulated in patient-derived fibroblasts, with a more pronounced reduction in P2 fibroblasts (Fig. 1D).

Fe-S clusters are essential for the enzymatic activity of aconitase. Aconitase catalyzes the conversion of citrate into isocitrate and is also related to iron metabolism [49]. Therefore, we assessed mitochondrial and cytosolic aconitase activity in both patient-derived and control fibroblasts. We observed reduced cytosolic aconitase activity in both lines in comparison to control and a reduced mitochondrial aconitase activity in P2 fibroblasts (Fig. 1E).

#### Mitochondrial dysfunction in FRDA fibroblasts: mitochondrial protein expression levels

**Fig. 2:**
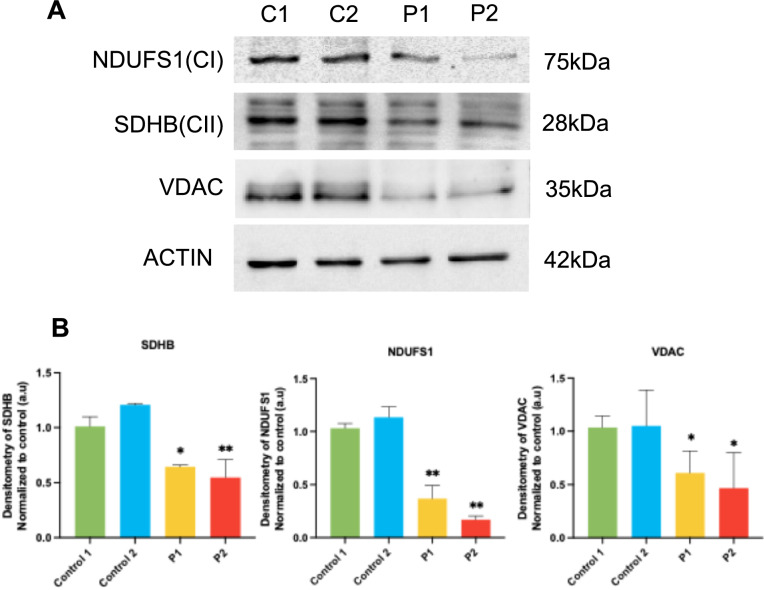
Mitochondrial subunits expression levels. **A** Immunoblotting analysis of cellular extracts from control (C1 and C2) and FRDA fibroblasts (P1 and P2). Protein extracts (15 μg) were separated on an SDS polyacrylamide gel and immunostained with antibodies against NDUFS1, SDHB and VDAC. Actin was used as a loading control. **B** Densitometry of the Western blot. Data represent the mean ± SD of three separate experiments. Data represent the mean ± SD of three separate experiments. Statistical significance between control and FRDA fibroblasts is expressed *p < 0.05 and **p < 0.01

Fe-S clusters also play a crucial role in the mitochondrial electron transport chain [50], since these clusters are contained in certain mitochondrial subunits and are essential for mitochondrial electron transport. We assessed the expression levels of mitochondrial complexes subunits such as NDUFS1, a complex I subunit, SDHB, a complex II subunit, and the mitochondrial mass marker VDAC by Western blot analysis. Our results showed that their levels were significantly reduced in FRDA fibroblasts compared to controls (Fig. 2A and B).

#### Altered expression levels of proteins related with iron metabolism

**Fig. 3:**
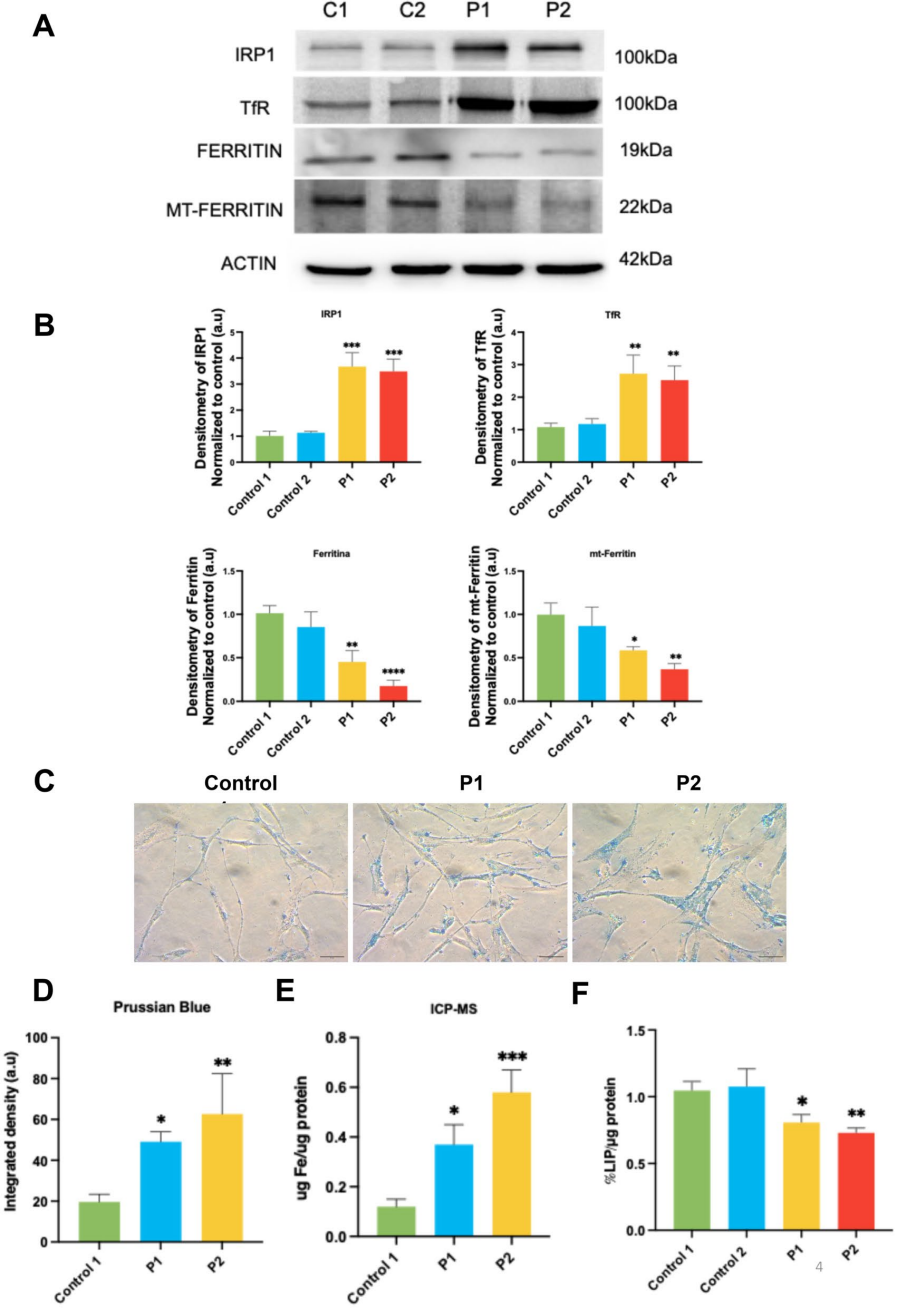
Expression levels of proteins involved in iron metabolism and iron accumulation. **A** Immunoblotting analysis of cellular extracts from control (C1 and C2) and FRDA fibroblasts (P1 and P2). Protein extracts (15 μg) were separated on an SDS polyacrylamide gel and immunostained with IRP1, TfR, Ferritin and mt-Ferritin. **B** Densitometry of Western blotting. **C** Representative images of iron accumulation determined by Prussian blue staining in control and FRDA fibroblasts (P1 and P2). Scale = 50μm. **D** Quantification of Prussian blue staining**. E** Quantification of iron levels in cell extracts by ICP-MS, normalized to μg of protein. **F** Graphical representation of labile iron levels quantified in control fibroblasts 1 and 2, and FRDA fibroblasts (P1 and P2) using the fluorometric assay based on calcein, following the protocol described in Materials and Methods. Data represent the mean ± SD of three independent experiments. Statistical significance between control and FRDA fibroblasts is expressed as *p < 0.05, **p < 0.01, ***p < 0.001 and ****p < 0.0001. A.U., arbitrary units

Considering that frataxin deficiency also contributes to the dysregulation of iron metabolism, we analyzed the expression levels of proteins involved in regulating cellular iron intake, export, and storage. Proteins such as IRP1 and TfR were overexpressed in FRDA fibroblasts, suggesting an increased iron influx into the cells. Conversely, cytosolic and mitochondrial ferritin were downregulated in FRDA fibroblasts, indicating insufficient iron availability for storage. (Fig. 3A and B).

#### Iron accumulation in FRDA fibroblasts

Iron metabolism dysregulation in FRDA promotes cellular iron accumulation, which is a hallmark of the disease [51]. We conducted two different techniques to determine cellular iron levels. Prussian blue staining showed that iron accumulation was more evident in FRDA fibroblasts compared to control fibroblasts (Fig. 3C and D). These results were further confirmed by ICP-MS, which revealed increased iron levels in P1 and P2 fibroblasts compared to control fibroblasts. Interestingly, iron levels were higher in P2 fibroblasts which show lower levels of FXN expression (Fig. 3E). Furthermore, to evaluate free iron availability, we performed a calcein assay to measure the labile iron pool (LIP). We observed that LIP was reduced in FRDA fibroblasts (Fig. 3F).

#### Lipofuscin accumulation and lipid peroxidation in FRDA fibroblasts: Bodipy C-11, Sudan black staining and autofluorescence

**Fig. 4:**
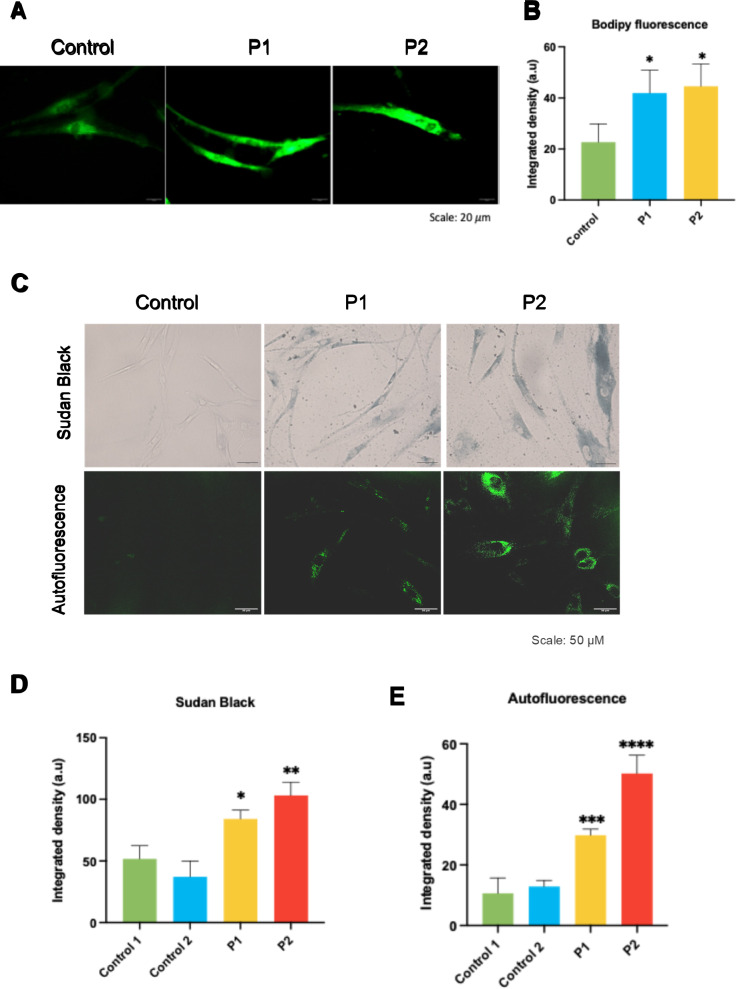
Determination of lipid peroxidation and lipofuscin accumulation in control and FRDA fibroblasts. **A** Representative images of lipid peroxidation in control and FRDA cells using BODIPY® 581/591 C11 staining. Scale bar = 20μm. **B** Fluorescence quantification of the oxidized form of BODIPY® C11. **C** Representative images of Sudan Black staining and autofluorescence obtained by confocal microscopy of control and FRDA fibroblasts (P1 and P2). Scale = 50μm. **D** Quantification of Sudan Black staining. **E** Quantification of autofluorescence. Data represent the mean ± SD of three separate experiments (30 cell images for each condition). Statistical significance between control and FRDA fibroblasts is expressed as *p < 0.05, **p < 0.01, ***p < 0.001 and ****p < 0.0001. A.U., arbitrary units

Since iron is a reactive element, cellular iron accumulation may trigger the formation of reactive oxygen species and cause oxidative stress, which leads to lipid peroxidation and the accumulation of lipofuscin granules [52]. These granules contain oxidized proteins, lipids, and metals. To determine lipid peroxidation levels, we used Bodipy C-11 labeling. We observed higher levels of fluorescence in FRDA fibroblasts compared to control fibroblasts (Fig. 4A and B), indicating increased lipid peroxidation.

Next, we used Sudan Black staining to determine lipofuscin accumulation. FRDA fibroblasts exhibited higher levels of lipofuscin compared to controls (Fig. 4C and D). Given that lipofuscin is an autofluorescent pigment, we confirmed these findings by measuring autofluorescence. FRDA fibroblasts displayed greater autofluorescence than control fibroblasts (Fig. 4C and E). Both results suggest that fibroblasts with *FXN* mutations show increased lipofuscin accumulation.

### Effect of ALA supplementation on pathophysiological alterations in FRDA fibroblasts and iNs.

#### Effect of ALA on frataxin protein and transcripts expression levels in FRDA fibroblasts

**Fig. 5:**
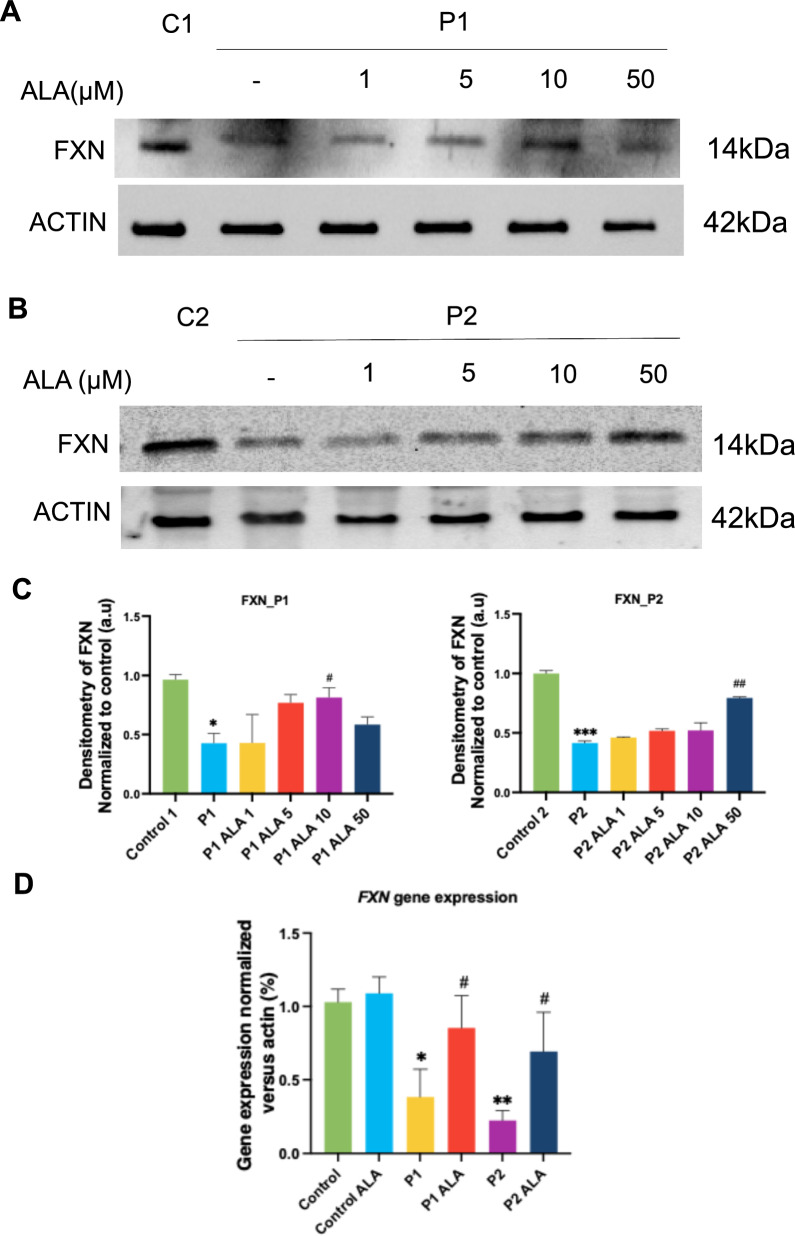
Dose–response curve of ALA on frataxin levels in FRDA fibroblasts. **A** Effect of ALA (1-50μM) on FXN expression in cell extracts from Control 1 and P1 fibroblasts. Actin expression levels were used as the loading control. **B** Effect of ALA (1-50μM) on FXN expression in cell extracts from Control 2 and P2 fibroblasts. Actin expression levels were used as the loading control. **C** Densitometry of frataxin expression levels. **D** Analysis of *FXN* gene expression by RT-qPCR in control and FRDA fibroblasts (P1 and P2), untreated and treated with ALA. The results are normalized to actin expression levels. Statistical significance between control and FRDA fibroblasts is represented as *p < 0.05 and **p < 0.01. Statistical significance between untreated and treated FRDA fibroblasts is expressed as ^#^*p* < 0.05, ^##^*p* < 0.01

Next, we evaluated the effect of ALA, a well-known antioxidant [53], on cell pathological alterations. First, we determined the most effective dose for each FRDA fibroblast cell line. For that purpose, we performed dose–response curves and measured frataxin expression levels by Western blot. After these screenings with different concentrations, we observed that 10 μM of ALA was the lowest concentration able to increase frataxin expression levels in P1 fibroblasts (Fig. 5A and C), while 50 μM was the most effective in P2 fibroblasts (Fig. 5B and C). These doses (10 μM for P1 and 50 μM for P2) were used in all subsequent experiments. Once these doses were established, we evaluated the effect of ALA on frataxin transcript expression levels by RT-qPCR. As shown in Fig. 5D, ALA treatment significantly increased transcript levels of frataxin in both FRDA fibroblast cell lines.

#### Effect of ALA on susceptibility to erastin-induced ferroptosis in FRDA fibroblasts

**Fig. 6:**
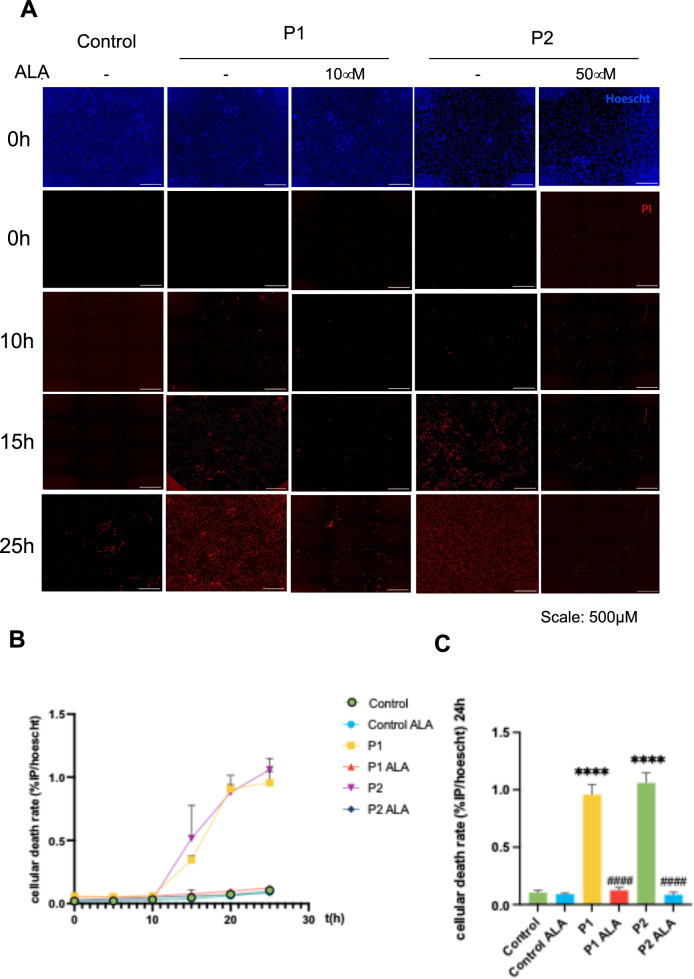
Effect of ALA on ferroptosis in control and FRDA fibroblasts. **A** Representative images of erastin-induced cell death at different time points in control and FRDA fibroblasts (P1 and P2), untreated and treated with ALA. Scale = 500μm. **B** Graphical representation of the cell death rate in control and FRDA fibroblasts (P1 and P2), untreated and pre-treated with ALA and exposed to erastin at 5 μM at different time points. **C** Graphical representation of the cell death rate induced by erastin in untreated and pre-treated fibroblasts with ALA at 24 h. The data represent the mean ± SD of three wells. Statistical significance between control and FRDA fibroblasts is represented as ****p < 0.0001. The statistical difference between untreated and treated fibroblasts is expressed as ^####^*p* < 0.0001

To investigate whether ALA could prevent cell death, we induced ferroptosis by treating cell culture with erastin for 24 h under different conditions: without previous treatment and previously supplemented with ALA (negative controls were also analyzed). Images were taken every hour, and we observed that after 15 h of incubation, erastin induced cell death in FRDA fibroblasts but not in control fibroblasts (Fig. 6A and B). We also calculated the cellular death rate after 24 h of erastin exposure (Fig. 6C). However, the sensitivity of FRDA fibroblasts pre-treated with ALA to erastin was significantly reduced, suggesting that ALA supplementation may protect FRDA fibroblasts against ferroptosis.

#### Effect of ALA on proteins related to ISC biosynthesis in FRDA fibroblasts.

**Fig. 7:**
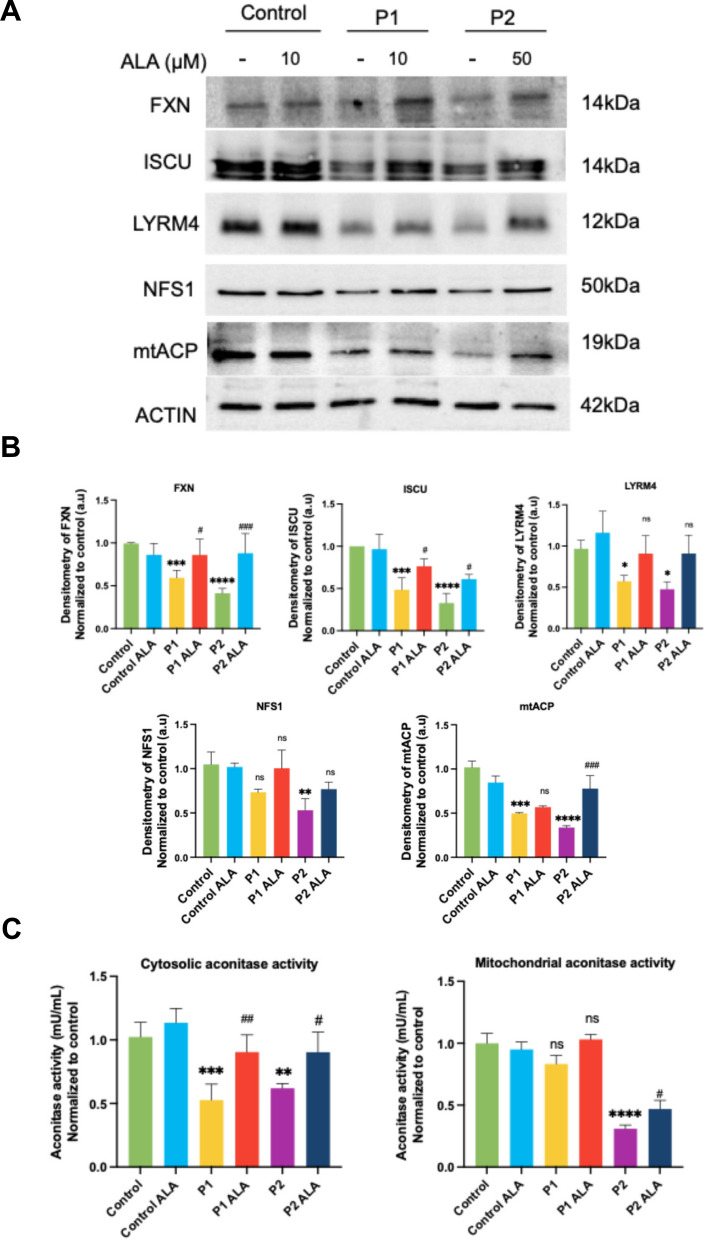
Effect of ALA supplementation on proteins involved in Fe-S cluster assembly and aconitase in FRDA fibroblasts. **A** Representative images of protein levels involved in Fe-S clusters in control and FRDA fibroblasts (P1 and P2), untreated and treated with ALA. Actin expression levels were used as the loading control. **B** Densitometry of Western blot. The results are normalized to the loading control and expressed as the mean ± SD of three independent experiments. **C** Quantification of mitochondrial and cytosolic aconitase using extracts from control and FRDA fibroblasts untreated and treated with ALA using a colorimetric assay. Statistical significance between control and FRDA fibroblasts is represented as *p < 0.05, **p < 0.01, ***p < 0.001, and ****p < 0.0001. Statistical significance between untreated and treated fibroblasts is expressed as ^#^*p* < 0.05, ^##^*p* < 0.01, ^###^p < 0.001, ns: not significant

Given that ALA was able to reduce the cellular death rate induced by erastin, we next analyzed the effect of ALA at the molecular level. We assessed expression levels of proteins involved in ISC biosynthesis, such as FXN, LYRM4, ISCU, NFS1 and mtACP before and after ALA supplementation. Western blot assays showed that ALA supplementation significantly increased FXN, ISCU and mtACP expression levels in FRDA fibroblasts and improved, but not significantly, LYRM4 and NFS1 expression levels (Fig. 7A and B). As mtACP is involved in lipoic acid biosynthesis [54], the positive effect of ALA was also corroborated by assessing the lipoylation levels of mitochondrial proteins by Western blotting and immunofluorescence (Fig. S1A, S1B, S1C and S1D). As anticipated, the decreased levels of protein lipoylation in FRDA fibroblasts improved significantly following ALA supplementation.

To go further, we hypothesized that these positive results were related to an improvement in ISC biosynthesis efficiency. To confirm this hypothesis, we determined aconitase activity. We observed that cytosolic aconitase activity significantly improved after supplementation of ALA in P1 and P2 fibroblasts. ALA also increased mitochondrial aconitase activity in P1 and P2 fibroblasts, although it was statistically significant only in P2 (Fig. 7C).

#### Effect of ALA on mitochondrial dysfunction in FRDA fibroblasts

**Fig. 8:**
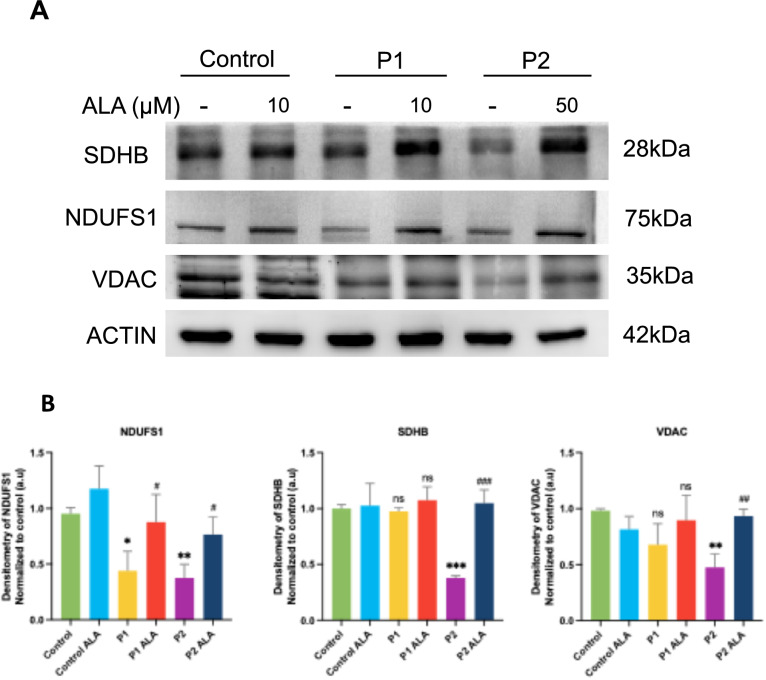
Effect of ALA supplementation on mitochondrial mass and mitochondrial subunits expression levels in FRDA fibroblasts. **A** Representative images of mitochondrial subunits expression levels in control and FRDA fibroblasts (P1 and P2), untreated and treated with ALA. Actin expression levels were used as the loading control. **B** Densitometry of Western blot. Data is represented as the mean ± SD of three independent experiments. Statistical significance between control and FRDA fibroblasts is represented as *p < 0.05, **p < 0.01, ***p < 0.001. Statistical significance between untreated and treated fibroblasts is expressed as ^#^*p* < 0.05, ^##^*p* < 0.01, ^###^*p* < 0.001, ns: not significant

Next, we examined the effect of ALA on mitochondrial protein expression levels and mitochondrial function. As is shown in Fig. 8A and B, ALA treatment increased expression levels of NDUFS1, SDHB and VDAC in P2, and NDUFS1 in P1 fibroblasts. Furthermore, although the expression levels of SDHB and VDAC in P1 fibroblasts also increased with treatment, this increase was not statistically significant. Altogether, those findings imply that ALA treatment has a positive impact on mitochondrial metabolism in FRDA fibroblasts.

Since we found an increase in VDAC expression, a marker for mitochondrial mass, after ALA treatment, we wondered if this supplementation could also induce mitochondrial biogenesis. To address this question, we studied the effect of ALA on key proteins involved in mitochondrial biogenesis, such as PGC1-α, P-PGC1-α, SIRT1, PPARγ and P-PPARγ. We found that expression levels of these proteins increased after ALA treatment in both P1 and P2 FRDA fibroblasts (Fig. S2A and B).

#### Effect of ALA on mitochondrial function in FRDA fibroblasts

**Fig. 9:**
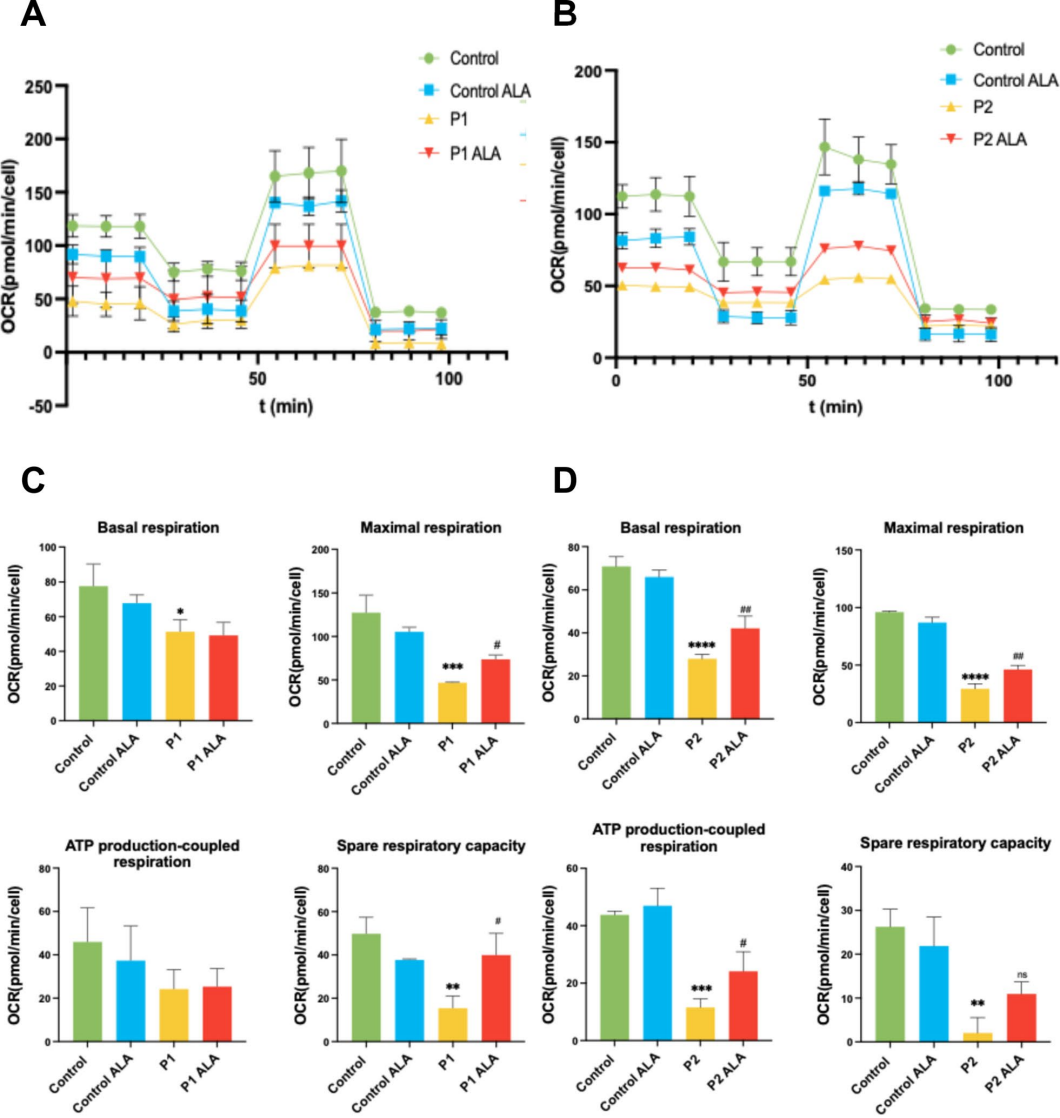
Effect of ALA on mitochondrial bioenergetics in control and FRDA fibroblasts. **A** Representative graph of the respiratory profile of control and P1 fibroblasts untreated and treated with ALA. OCR was measured under basal conditions, and after sequential injection of oligomycin, FCCP, rotenone, and antimycin A in control and P1 fibroblasts, untreated and treated with ALA. Each point represents an OCR measurement and is expressed as the mean ± SD. **B** Representative graph of the respiratory profile of control and P2 fibroblasts untreated and treated with ALA. OCR was measured under basal conditions, and after sequential injection of oligomycin, FCCP, rotenone, and antimycin A in control and P2 fibroblasts, untreated and treated with ALA. Each point represents an OCR measurement and is expressed as the mean ± SD. **C** Basal respiration, maximal respiration, reserve respiratory capacity, and ATP-linked respiration were calculated to determine the effect of ALA in P1 fibroblasts. **D** Basal respiration, maximal respiration, reserve respiratory capacity, and ATP-linked respiration were calculated to determine the effect of ALA in P2 fibroblasts. The results are expressed as the mean ± SD of three independent experiments. Statistical significance between control and P1 fibroblasts is represented as **p < 0.01, ***p < 0.001. Statistical significance between untreated and treated fibroblasts is expressed as ^#^*p* < 0.05, ns: not significant

To study the effect of ALA on mitochondrial function, we determined oxygen consumption rate using a Seahorse XFe24 analyzer. After treating FRDA fibroblasts with ALA, OCR was increased in FRDA fibroblasts (Fig. 9A and B). These improvements could be a consequence of increased mitochondrial protein expression and enhanced ISC biosynthesis. Figure 9A and B show the respiratory profile of control and P1 and P2 fibroblasts untreated and treated with ALA. Supplementation with ALA improved mitochondrial parameters such as maximal respiration and spare respiratory capacity in P1 fibroblasts (Fig. 9C) and basal respiration, maximal respiration and ATP production-coupled respiration in P2 fibroblasts (Fig. 9D).

#### Effect of ALA on lipid peroxidation, and iron and lipofuscin accumulation in FRDA fibroblasts

**Fig. 10:**
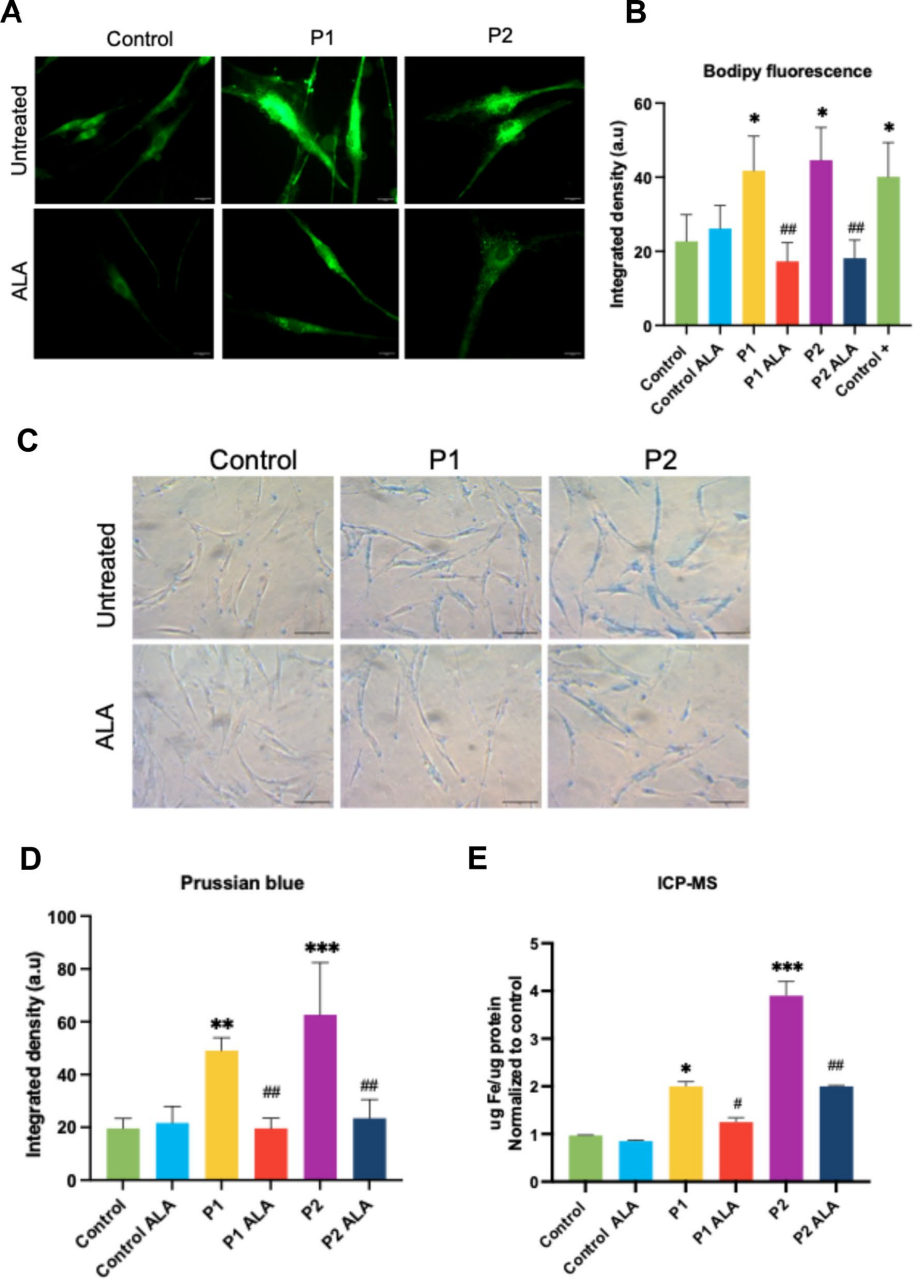
Effect of ALA on lipid peroxidation and iron accumulation in control and FRDA fibroblasts. **A** Representative images of lipid peroxidation in control and FRDA fibroblasts untreated and treated with ALA using BODIPY® 581/591 C11 staining. Control cells treated with Luperox® (500 μM) for 15 min were used as a positive control of lipid peroxidation. Scale bar = 20μm. **B** Fluorescence quantification of the oxidized form of BODIPY® C11. Data represent the mean ± SD of three separate experiments. **C** Representative images of Prussian blue staining in control and FRDA fibroblasts untreated and treated with ALA. Scale bar = 50μm. **D** Quantification of Prussian blue staining. **E** Quantification of iron levels in cell extracts by ICP-MS, normalized to μg of protein. The data represent the mean ± SD of three independent experiments. Statistical significance between control and FRDA fibroblasts is expressed as *p < 0.05, **p < 0.01 and ***p < 0.001. Statistical significance between untreated and treated fibroblasts is expressed as ^#^*p* < 0.05 and ^##^*p* < 0.01, A.U., arbitrary units

Lipid peroxidation and iron accumulation have been described as the main hallmarks of the disease. Therefore, we decided to study the effect of ALA on these pathophysiological features. Bodipy assay showed that ALA supplementation significantly reduced lipid peroxidation on FRDA fibroblasts (Fig. 10A and B). Additionally, methods such as Prussian blue staining and ICP-MS assays revealed that ALA supplementation reduced iron accumulation in both cell lines (Fig. 10C, D and E).

**Fig. 11:**
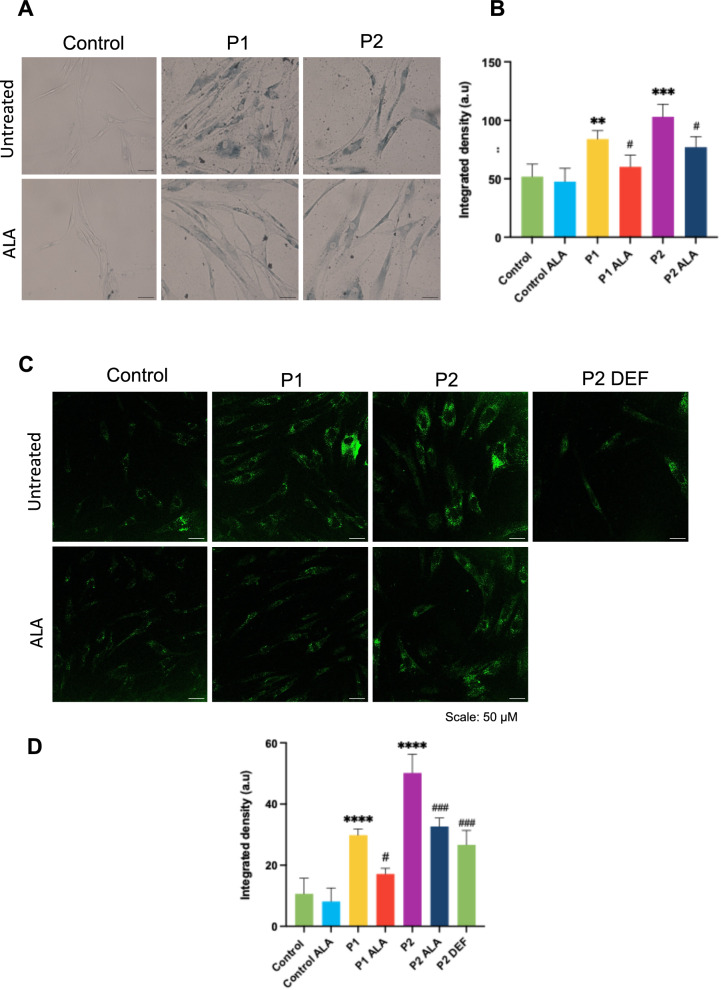
Effect of ALA on lipofuscin accumulation in control and FRDA fibroblasts. **A** Representative images of Sudan Black staining in control and FRDA fibroblasts untreated and treated with ALA. Scale bar = 50 μm. **B** Quantification of Sudan Black. **C** Representative images obtained by confocal microscopy of the autofluorescence of control and FRDA fibroblasts untreated and treated with ALA. Deferiprone was added to P2 fibroblasts to demonstrate that iron accumulation promotes lipofuscin aggregates. Scale bar = 50 μm. **D** Data represents the mean ± SD of three independent experiments. Statistical significance between control and FRDA fibroblasts is expressed as **p < 0.01, ***p < 0.001 and ****p < 0.0001. Statistical significance between untreated and treated fibroblasts is expressed as ^#^*p* < 0.05, ^###^*p* < 0.001, A.U., arbitrary units

**Fig. 12:**
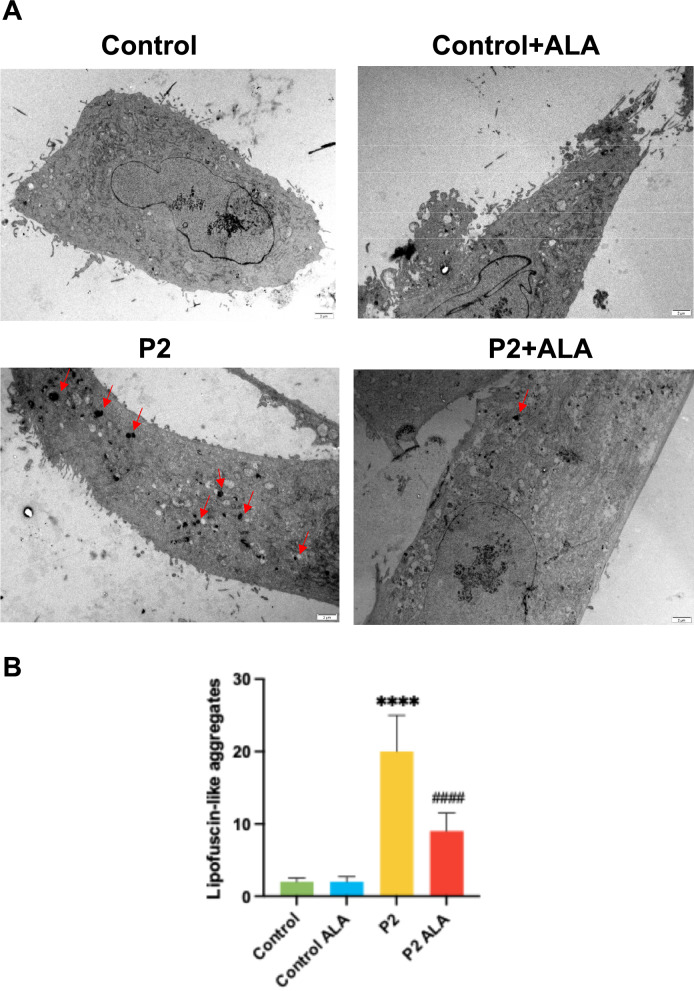
Effect of ALA on lipofuscin-like aggregates in control and P2 fibroblasts. **A** Representative images of TEM in control and P2 fibroblasts untreated and treated with ALA. Scale bar = 2μm. **B** Quantification of lipofuscin-like aggregates. Data represent the mean ± SD of the examination of 50 cells per condition. Statistical significance between control and P2 fibroblasts is expressed as ****p < 0.0001. Statistical significance between untreated and treated fibroblasts is expressed as ^####^*p* < 0.0001

As we observed in Sudan Black staining and autofluorescence assay, the reduction in lipid peroxidation and iron accumulation led to a decrease in lipofuscin accumulation determined by Sudan Black staining (Fig. 11A and B) and autofluorescence (Fig. 11C and D). We also observed a high number of lipofuscin-like aggregates per cell in P2 fibroblasts using transmission electron microscopy that were significantly reduced after ALA treatment (Fig. 12A and B). Interestingly, TEM analysis also revealed that mitochondrial morphology in FRDA cells showed mitochondrial vacuolization and condensation of mitochondrial compounds forming dense lipofuscin-like granules (Fig. S3, S4 and S5).

#### Effect of ALA on cellular antioxidant system

One of the main factors leading to ferroptosis in FRDA fibroblasts is the deficiency of the antioxidant system, which leads to high levels of oxidative stress. To assess the enzymatic antioxidant system and the impact of ALA on them, we performed Western blot assays to measure the expression levels of antioxidant enzymes such as GPX4, SOD1 and catalase. We also determined the expression levels of NRF2 transcription factor which regulates the expression of antioxidant enzymes. As is shown in Fig. S6A and B, antioxidant enzymes and NRF2 expression were downregulated in FRDA fibroblasts with respect to control. After ALA supplementation, these antioxidant protein expression levels were significantly increased.

#### ALA induces frataxin expression through PPARy activation in FRDA fibroblasts

**Fig. 13:**
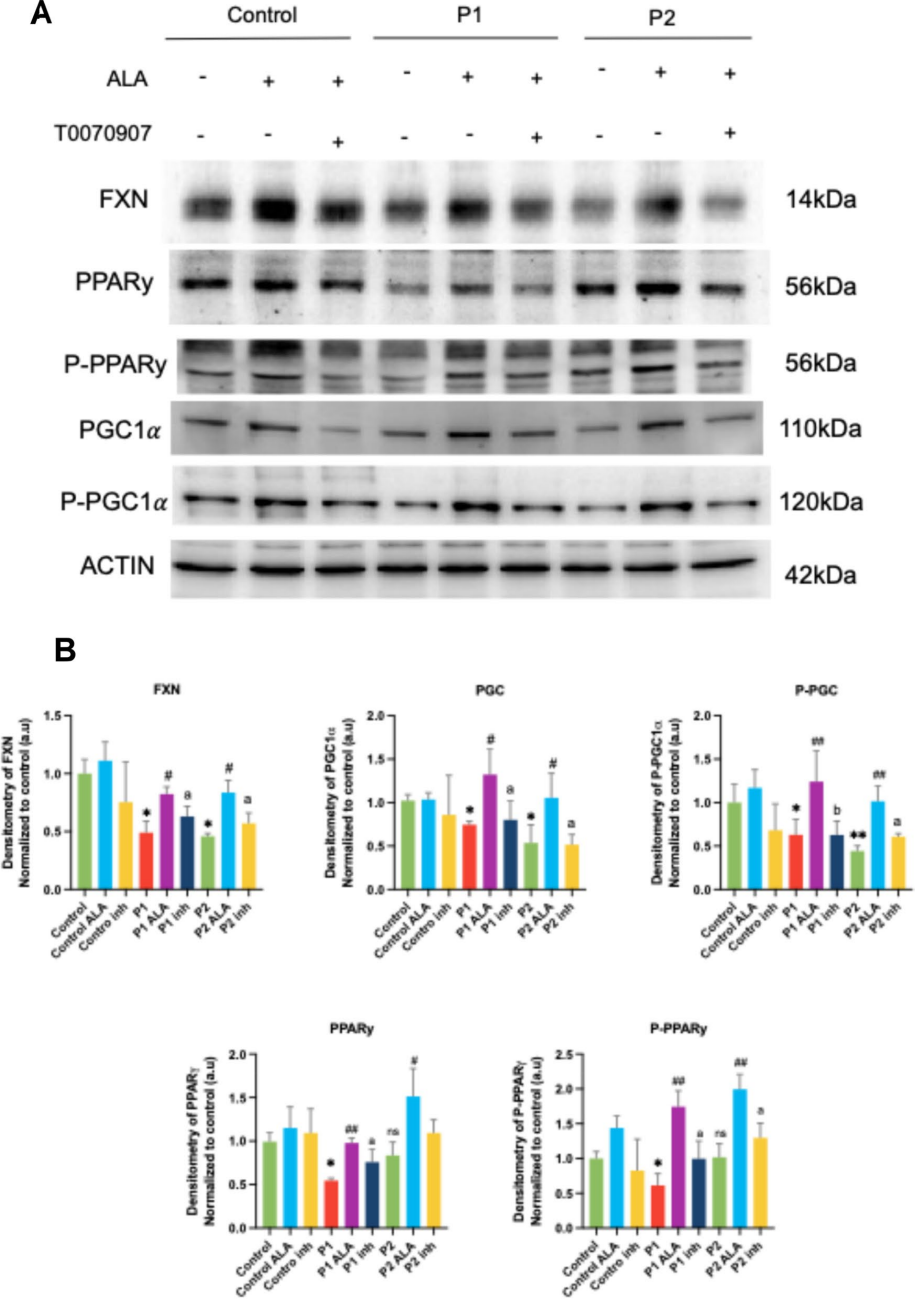
Effect of ALA and T0070907, a PPARγ inhibitor, supplementation on expression levels of key proteins in FRDA fibroblasts. **A** Representative images of protein expression levels (FXN, PPARγ, P-PPARγ, PGC1α and P-PGC1α) in control and FRDA fibroblasts (P1 and P2), both untreated and treated with ALA and following simultaneous supplementation with T0070907 at 6 μM and ALA. Actin expression levels were used as the loading control. **B** Densitometry of Western blot. The results are normalized to the loading control and expressed as the mean ± SD of three independent experiments. Statistical significance between control and FRDA fibroblasts is represented as *p < 0.05, **p < 0.01. Statistical significance between untreated and treated fibroblasts is expressed as ^#^*p* < 0.05 and ^##^*p* < 0.01. Statistical significance between ALA-treated and T0070907 and ALA-treated fibroblasts is expressed as ^a^*p* < 0.05 and ^b^*p* < 0.01

To further investigate if mitochondrial biogenesis was a key mechanism for inducing frataxin expression, we used a selective PPARy inhibitor, T000070907. We used 2μM and 6μM in P1 and P2 fibroblasts, respectively, in order to inhibit PPARy activation. The addition of T000070907 prevented the ALA-induced increase in frataxin expression levels. (Fig. 13A and B). We also observed that supplementation with T000070907 prevented the ALA-induced increase of PPARγ, P-PPARγ, PGC1α and P-PGC1α expression levels. These results suggested that ALA plays an important role in the PPARγ activation and, consequently, inducing mitochondrial biogenesis in FRDA fibroblasts.

#### Effect of ALA on FRDA iNs

To further study FRDA in neuronal cell models, we transdifferentiated FRDA and control fibroblasts into iNs by direct reprogramming. This technique allowed us to study the disease in one of the most affected cell types. Control and FRDA fibroblasts were infected with lentiviral vectors containing proneural genes and the REST complex. After 27 days, fibroblasts were converted into iNs showing a typical neuron-like morphology and positive immunoreactivity against Tau, a neuron-specific protein.

We evaluated two parameters related to this technique, conversion efficiency and purity. We found different values of these parameters for each fibroblast line. While control cells showed a conversion efficiency of 25%, P1 and P2 showed efficiencies of 40% and 10%, respectively. The purity of the control iNs culture was 50%, compared to 60% for P1 iNs and 80% for P2 iNs.

**Fig. 14:**
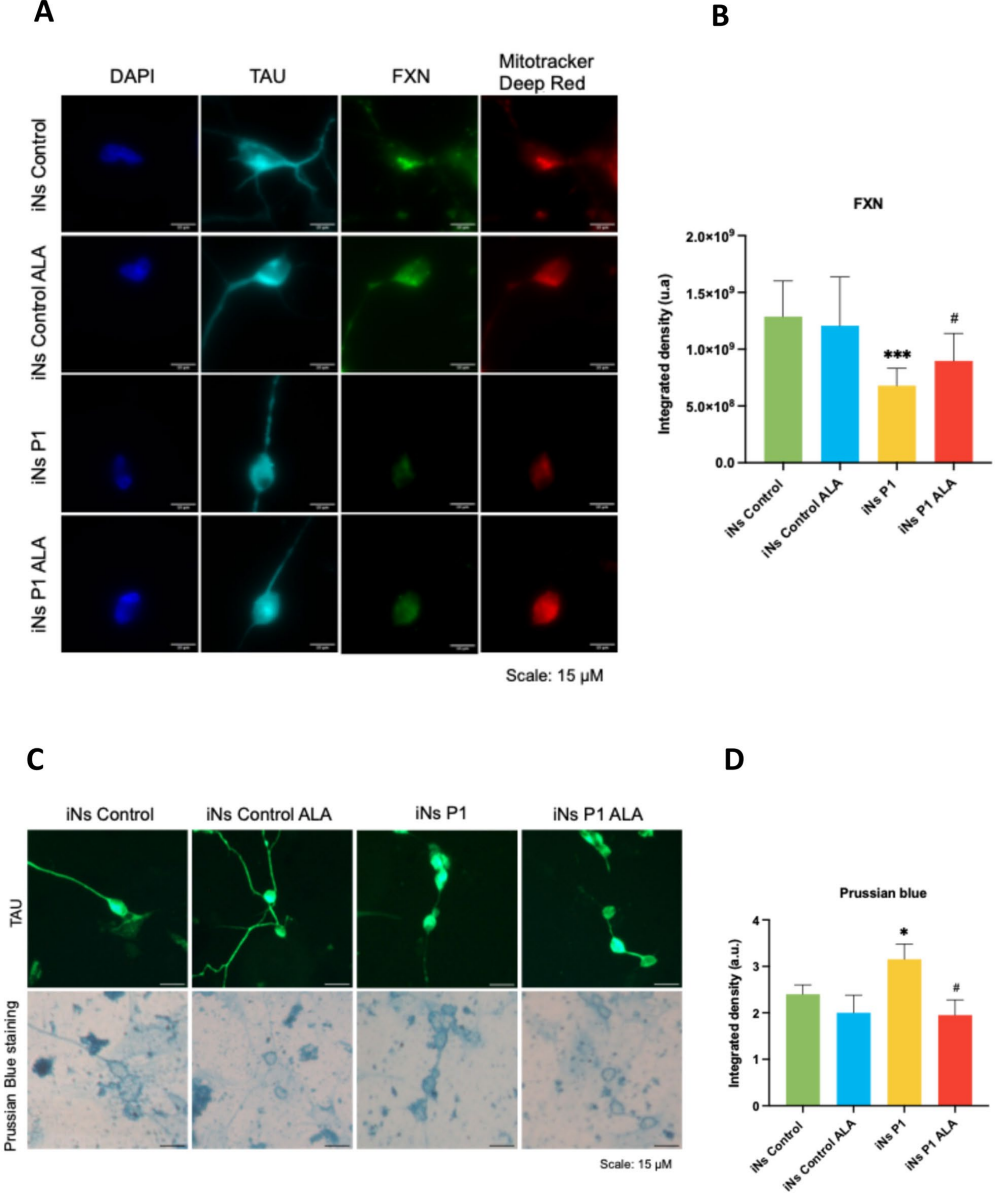
Effect of ALA on FXN expression and iron accumulation in P1 iNs. **A** Representative images of FXN fluorescence intensity in TAU + cells. Scale = 15μm. **B** Fluorescence intensity of FXN in control and P1 iNs, untreated and treated with 10μM ALA. **C** Representative images of Prussian blue staining in control and FRDA iNs in TAU + cells, untreated and treated with 10 μM ALA. Scale = 15 μm. **D** Quantification of iron accumulation in control and P1 iNs, untreated and treated with 10μM ALA. The data represent the mean ± SD of three experiments. Statistical significance between control and P1 iNs is represented as *p < 0.05 and ***p < 0.001. The statistical difference between untreated and ALA-treated iNs is represented as ^#^*p* < 0.05

**Fig. 15:**
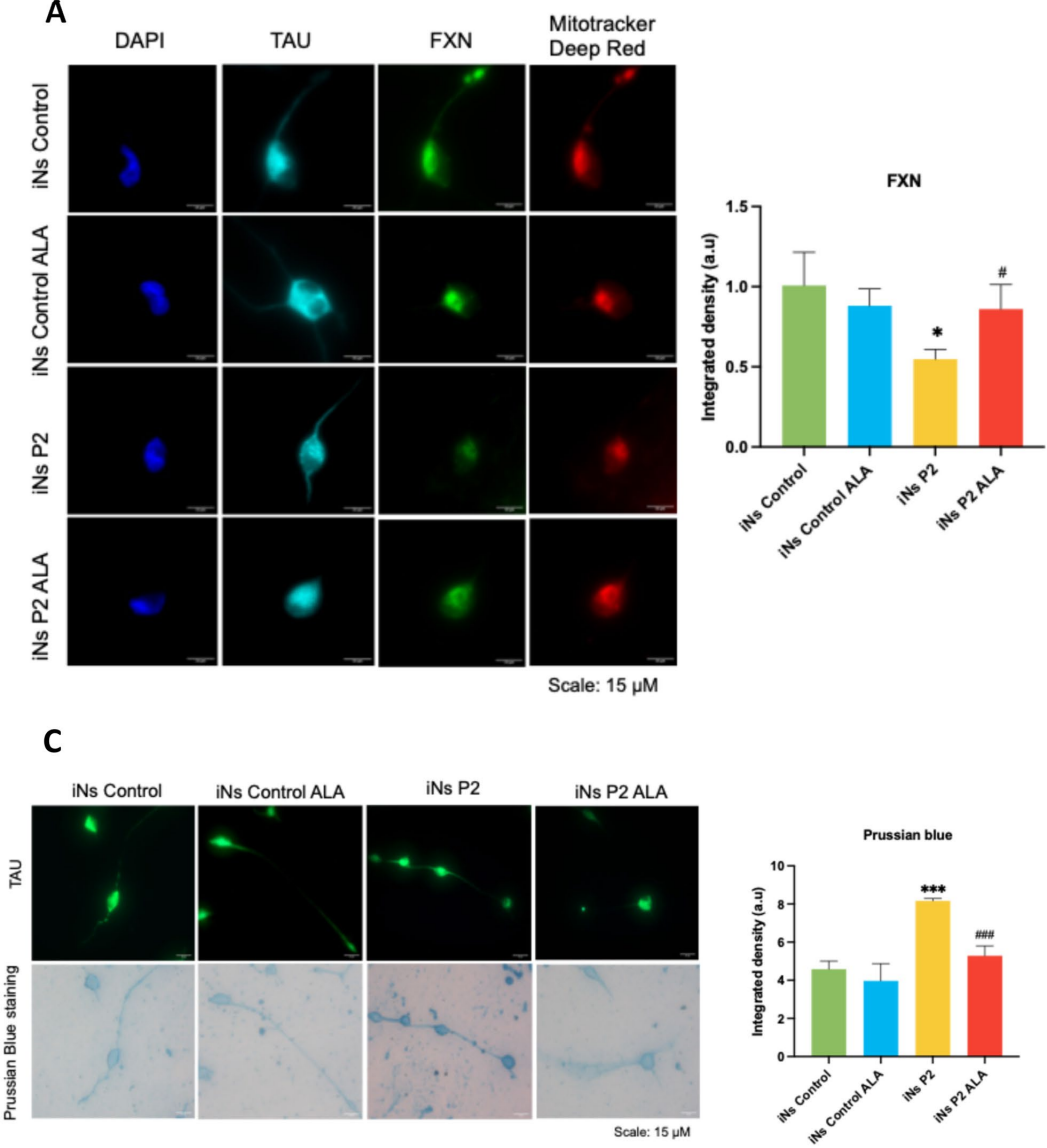
Effect of ALA on FXN expression and iron accumulation in P2 iNs. **A** Representative images of FXN fluorescence intensity in TAU + cells. Scale = 15μm. **B** Fluorescence intensity of FXN in control and P2 iNs, untreated and treated with 50μM ALA. **C** Representative images of Prussian blue staining in control and in TAU + cells, untreated and treated with 50 μM ALA. Scale = 15 μm. **D** Quantification of iron accumulation in control and P2 iNs, untreated and treated with 50 μM ALA. Data is expressed as the mean ± SD of three experiments. Statistical significance between control and P2 iNs is represented as *p < 0.05 and ***p < 0.001. The statistical difference between untreated and ALA-treated iNs is represented as ^#^*p* < 0.05 and ^###^*p* < 0.001

Next, we studied this cellular model by examining the main pathophysiological features observed in FRDA fibroblasts, specifically frataxin expression and iron accumulation, and the effect of ALA on these iNs. Figures 14A and 15A show that frataxin protein expression was reduced in P1 and P2 iNs, which was restored after 10 μM and 50 μM of ALA treatment, respectively. Regarding iron accumulation, Prussian blue staining revealed an increase in P1 and P2 iNs, which was reduced following ALA supplementation (Figs. 14B and 15B).

## Discussion

FRDA is an autosomal recessive disorder mainly caused by the expansion of the GAA codon in the first intron of the *FXN* gene. The trinucleotide repeats in FRDA patients can reach over 1000 GAA copies reducing frataxin expression [32]. It has been described that gene therapy using exogenous frataxin can reverse FDRA symptoms in an *FXN knock-out* mouse model, increasing life expectancy and Fe-S bioavailability, which indicates that its pathophysiology is directly related to frataxin deficiency [55, 56].

In this study, we evaluated the pathological alterations in two cellular models of FRDA: patient-derived skin fibroblasts, and iNs obtained by direct reprogramming. When we assessed *FXN* expression levels by RT-qPCR and Western blot in FRDA fibroblasts, we found that expression of this gene was reduced in both cell lines and it was even more severe in the case of P2, which harbors a higher number of GAA repeats.

One of the main consequences of frataxin deficiency is the dysregulation of Fe-S cluster biosynthesis, which affects the expression levels of proteins involved in this process [57]. Consequently, aconitase activity is compromised in both FRDA fibroblast cell lines with respect to control. Cytosolic aconitase activity was significantly reduced in both FRDA fibroblast cell lines. This cytosolic isoform of the bifunctional enzyme may function as IRP1 in response to low cellular iron levels, which could explain why it is significantly reduced in both fibroblast lines. Previous studies have reported reduced aconitase activity in a FRDA mouse model as a direct consequence of frataxin deficiency [7, 58].

Given that both frataxin and aconitase are closely related to iron metabolism [59], protein expression levels involved in iron metabolism were also altered. Expression levels of proteins such as IRP1 and TfR were increased in FRDA fibroblasts, while ferritin and mitochondrial ferritin were downregulated, suggesting a deficiency in intracellular iron availability. Low ferritin levels were consistent with reported studies [60, 61]. However, other works found that ferritin and mitochondrial ferritin levels were increased in FRDA models [62]. In FRDA patients’samples, histological analysis suggested that mitochondrial ferritin might be involved in the formation of the iron-rich structures [63]. However, data obtained in mice models suggested that mitochondrial ferritin is not involved since iron was reported to be mostly present as mineral non-ferritin aggregates [60].

Interestingly, Calcein-AM assay indicated a reduction in free iron, even though FRDA fibroblasts showed intracellular iron accumulation. Furthermore, our results indicated that iron accumulation was higher in P2 than in P1 fibroblasts, which correlates with a greater alteration in iron metabolism in this cell line. Several studies indicated that iron deposits were found inside the mitochondrial matrix [26, 27, 30]. However, there are discrepancies regarding mitochondrial iron accumulation in FRDA fibroblasts [14].

Although oxidative stress and iron accumulation are hallmark features of FRDA, it is unclear whether iron accumulation is a cause or a consequence of the disease [15]. Some studies using yeast models suggest that dysregulation of iron homeostasis is the first event observed after frataxin expression is repressed, followed by iron accumulation and decreased aconitase activity [64, 65]. In contrast, Seznec et al. observed that in the Frda/MCK mouse model, the initial phenotype after frataxin repression was the downregulation of Fe-S-dependent enzymes, which was then followed by iron accumulation [62]. Several researchers additionally propose the hypothesis of a feedback loop linking oxidative stress with iron accumulation [66, 67].

In a previous study by our group, we also demonstrated the existence of a vicious circle involving iron accumulation, lipofuscin buildup, and lipid peroxidation [52, 68]. Iron accumulation promotes the production of ROS through the Fenton reaction, leading to lipid peroxidation. In turn, lipid peroxidation induces mitochondrial dysfunction and the formation of iron-rich lipofuscin aggregates [69]. Interestingly, we observed that lipofuscin aggregates in FRDA fibroblasts were formed inside mitochondria. Previous observations in various cellular models of neurodegenerative disorders have also suggested a mitochondrial source for lipofuscin [70, 71].

Due to the Fe-S cluster deficiency, we assessed both the expression and activity of mitochondrial complexes that contain Fe-S clusters, such as Complex I and Complex II [50, 72]. Both FRDA fibroblast cell lines showed reduced expression levels of the mitochondrial subunits, NDUFS1 and SDHB compared to control, which could be explained by the Fe-S cluster deficit. Another plausible explanation for disruptions in mitochondrial subunits expression levels could be the reduced mitochondrial mass. However, a work from Davide D. et al. showed reduced levels of NDUFS1 and SDHB in mitochondrial extracts, while the expression levels of other mitochondrial subunits in Complexes I, II and III remained normal [73]. Recent studies have proven that frataxin is enriched in mitochondrial cristae under normal conditions, while in FRDA patient-derived cells, frataxin is located in the mitochondrial matrix [74]. In addition, frataxin binds to complexes I, II and III in healthy cells. These findings suggest that frataxin could impact on mitochondrial Complex activity [73, 75].

These mitochondrial alterations were associated with mitochondrial dysfunction affecting parameters such as basal respiration, ATP-coupled respiration, or spare respiratory capacity. These findings are validated by several studies that confirm mitochondrial dysfunction in FRDA iPSC-derived neurons [76] and neuron-like cells derived from rats [77].

It is important to emphasize the impact of mtACP downregulation in FRDA fibroblasts. In addition to its involvement in Fe-S cluster byosinthesis [78], mtACP is also an integral component of mitochondrial respiratory Complex I and is required for its proper assembly and function [79, 80]. Its downregulation may exacerbate the effect of FXN deficiency on Complex I activity. Furthermore, mtACP participates in type II mitochondrial fatty acid synthesis, a pathway necessary to produce octanoic acid, the precursor for the novo lipoic acid biosynthesis. Accordingly, mtACP deficiency is known to hinder lipoic acid biosynthesis via this pathway [54], and we observed reduced levels of lipoylated proteins in FRDA fibroblasts.

As a therapeutic strategy, we examined the effect of ALA treatment on the pathophysiology of FRDA fibroblasts. Our results showed that ALA supplementation improved frataxin expression and cell pathophysiology, avoiding induced cellular death and therefore, suggesting its potential as a therapeutic approach in FRDA.

ALA is an essential cofactor for several mitochondrial enzyme complexes. However, exogenous ALA supplementation does not result in effective incorporation into lipoylated proteins in humans [81]. Thus, ALA must be synthesized de novo within mitochondria using intermediates from mitochondrial fatty acid synthesis, S-adenosylmethionine and Fe-S clusters as cofactors [82]. Therefore, the positive effects of ALA supplementation on FRDA fibroblasts may be mainly mediated through its antioxidant or signaling properties.

Several studies suggest that ALA is an agonist of PPARγ, promoting increased mitochondrial biogenesis, OCR, and the expression of transcription factors such as PGC1α and NRF2 [83, 84]. Furthermore, it has been described that ALA induces mitochondrial biogenesis by increasing the SIRT1 and PGC1α expression, which have neuroprotective properties, reducing oxidative stress, inflammation and preventing cell death [85, 86].The inhibition of PPARγ using T0070907 [87, 88], avoided the beneficial effect of ALA, suggesting that PPARγ activation is at least one of the key mechanisms through which ALA ameliorates the mutant phenotype of FRDA fibroblasts. Therefore, ALA could have an indirect effect on mitochondrial function by increasing the expression of mitochondrial genes such as *FXN* and correcting the expression levels of mitochondrial proteins involved in ISC biosynthesis, including mtACP.

On the other hand, a recent work of Zhao et al*.* demonstrated that ALA reduced ferroptosis by activating the PPARγ/NRF2/GPX4 pathway in a mouse model of unexplained recurrent pregnancy loss [35]. Other studies showed that PPARγ stimulates the NRF2/ARE axis to counteract oxidative stress and ferroptosis [89]. In our study, we found that ALA also protects FRDA fibroblasts from erastin-induced death by reducing iron accumulation, lipid peroxidation and lipofuscin buildup, while increasing the expression of antioxidant proteins such as catalase, SOD1 and GPX4 and the expression of NRF2, a key regulator of stress response genes. These findings are consistent with previous studies reporting that ALA alleviates oxidative stress, ferroptosis and tau hyperphosphorylation in animal models of tauopathy mouse and Pakinson’s disease [90–93]. Taken together, these results suggest that ALA may play a key role in enhancing the antioxidant capacity of the cells and reducing their susceptibility to death by ferroptosis.

Fibroblasts have proven to be a useful and reliable model for studying the underlying mechanisms of FRDA. However, to further explore the pathophysiology of the disease, we investigate it in a more relevant cellular context, specifically neurons. Several neuronal models derived from mice or rats have been established, but iNs offer a unique advantage, as they allow us to study the pathophysiology of the disease in a neuronal model that maintains the same genetic mutation and epigenetics makers as the patient [40, 94, 95].

In this work, we focused on two key aspects of FRDA physiopathology in iNs: frataxin expression levels and iron accumulation. Our results showed that frataxin expression was significantly reduced in FRDA iNs in comparison to control iNs as we observed previously in FRDA fibroblasts, indicating that this model can be useful for studying the underlying mechanisms of the disease and for drug screening. Similarly, iPSCs-derived neurons from FRDA fibroblasts also exhibited reduced *FXN* expression compared to control [96].

The FRDA iNs model also showed iron accumulation, determined by Prussian blue staining. While some studies using iNs and iPSCs-derived neuronal models have reported deficiencies in Fe-S assembly proteins, oxidative stress, and neuronal death, there is limited research on iron accumulation in these neuronal models [97, 98]. Furthermore, we confirmed that ALA supplementation increased frataxin expression and reduced iron accumulation in FRDA iNs, concluding that this compound could be a potential therapeutic treatment for FRDA.

## Conclusion

In this study, we show that ALA supplementation can improve the pathological alterations in cellular models of FRDA by a mechanism involving the up-regulation of *FXN* transcription and protein expression levels.

Although ALA has a powerful antioxidant effect, its capability to activate PPARy and mitochondrial biogenesis is essential to restore frataxin expression in fibroblasts derived from FRDA patients. Further studies and controlled clinical trials are required to assess the clinical benefit of ALA in FRDA.

## Supplementary Information

Additional file1 (DOCX 4818 KB)

## Data Availability

Data supporting the findings of this study are not openly available due to reasons of sensitivity and to protect the privacy of individuals; however, data are available from the corresponding author upon reasonable request. Data are located in controlled access data storage at Pablo de Olavide University (https://jazmin.upo.es/bscw/bscw.cgi).

## Abbreviations

ALA: α-Lipoic acid
CNS: Central nervous system
DAPI: 4 ‘,6-Diamidino-2-fenilindol
DRG: Dorsal root ganglia
FRDA: Friedreich ataxia disease
FXN: Frataxin
GPX4: Glutathione peroxidase 4
GSH: Glutathione synthetase
ICP-MS: Inductively coupled plasma mass spectroscopy
iNs: Induced neurons
ISCs: Iron-sulfur clusters
ISCU: Iron-sulfur cluster assembly enzyme
IRP1: Iron regulatory proteins 1
LIP: Labile iron pool
LYRM4: LYR Motif Containing 4
MELAS: Mitochondrial encephalopathy, lactic acidosis and stroke-like episodes
mtACP: Mitochondrial acyl carrier protein
NFS1: NFS1 cysteine desulfurase
NRF2: Nuclear factor erythroid 2-related factor 2
OCR: Oxygen consumption rate
PGC1α: Transcriptional co-activator peroxisome proliferator-activated receptor gamma coactivator 1 alpha
P- PGC1α: Phospho-transcriptional co-activator peroxisome proliferator-activated receptor gamma coactivator 1 alpha
PLAN: PLA2G6-associated neurodegeneration
PKAN: Pantothenate kinase-associated neurodegeneration
PPARγ: Peroxisome proliferator-activated receptor Gamma
P- PPARγ: Phospho-peroxisome proliferator-activated receptor Gamma
REST: RE1-silencing transcription factor
RNS: Reactive nitrogen species
ROS: Reactive oxygen species
SIRT1: NAD-dependent protein deacetylase sirtuin-1
SOD1: Superoxide dismutase 1
TEM: Transmission electron microscopy
TfR: Transferin receptor
VDAC: Voltage-dependent anion channel
