# Supplementary material for: Alpha–lipoic acid supplementation improves pathological alterations in cellular models of Friedreich ataxia
Source: Orphanet J Rare Dis. 2025 Aug 23;20:453. doi: 10.1186/s13023-025-03990-z (PMC12374286; doi:10.1186/s13023-025-03990-z)
Supplement: Supplementary file 1 — Additional file1 (DOCX 4818 KB) [file 13023_2025_3990_MOESM1_ESM.docx]

**A**

**B**


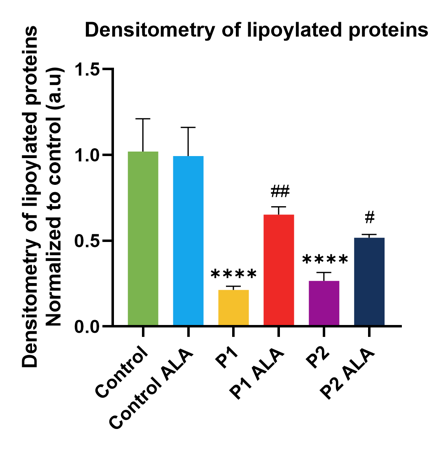
**
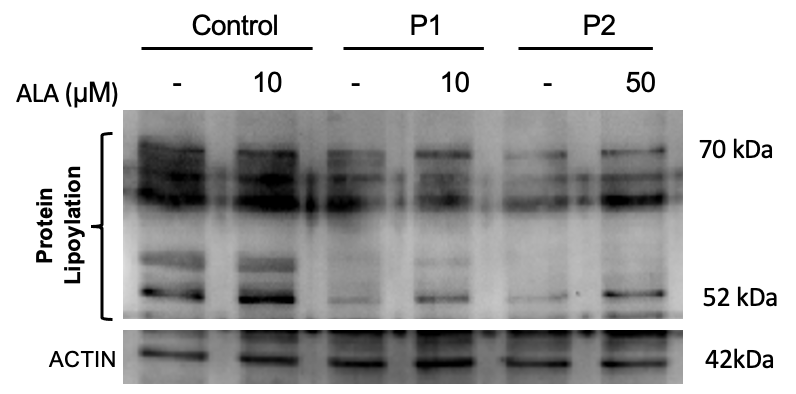
**

**C**

**D**

**Figure Supplementary 1. Effect of ALA supplementation on mitochondrial protein lipoylation. (A)** Western blot analysis of lipoylated proteins in control and FRDA fibroblasts (P1 and P2), untreated and treated with ALA. **(B)** Densitometry of Western blot. **(C)** Immunofluorescence of mitochondrial protein lipoylation. Cells were fixed and immunostained with the anti-ALA antibody. The signal from TOMM20 antibodies were used as a mitochondrial marker and nuclei were visualized with DAPI staining. Scale bar: 20 µm. **(D)** Quantification of fluorescence intensity of the lipoic acid antibody. Images were analyzed by ImageJ software (at least 30 images were taken and analyzed from each condition and experiment). Data is expressed as the mean ± SD of three independent experiments. Statistical significance between control and FRDA fibroblasts is represented as **p<0.01, and ****p<0.0001. Statistical significance between untreated and treated fibroblasts is expressed as ^#^p<0.05, ^##^p<0.01, ^###^p< 0,001.

**
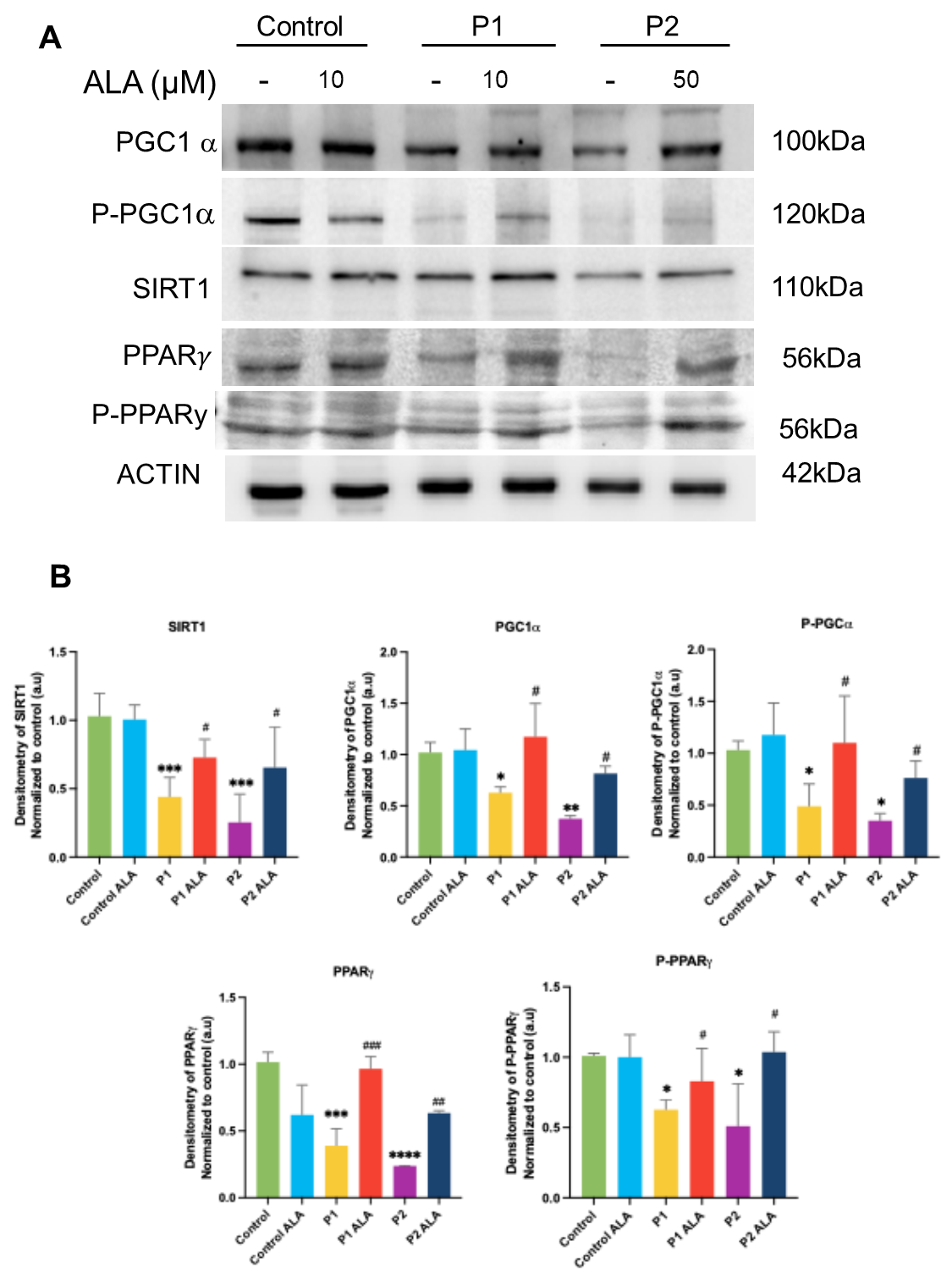
**

**Figure Supplementary 2. Effect of ALA supplementation on proteins involved in mitochondrial biogenesis in FRDA fibroblasts. (A)** Representative images of protein levels involved in mitochondrial biogenesis, PGC1α, P-PGC1α, SIRT1, PPARγ and P-PPARγ, in control and FRDA fibroblasts (P1 and P2), untreated and treated with ALA. Actin expression levels were used as the loading control. **(B)** Data is expressed as the mean ± SD of three independent experiments. Statistical significance between control and FRDA fibroblasts is represented as *p<0.05, **p<0.01, ***p<0.001, and ****p<0.0001. Statistical significance between untreated and treated fibroblasts is expressed as ^#^p<0.05, ^##^p<0.01, ^###^p<0.001.


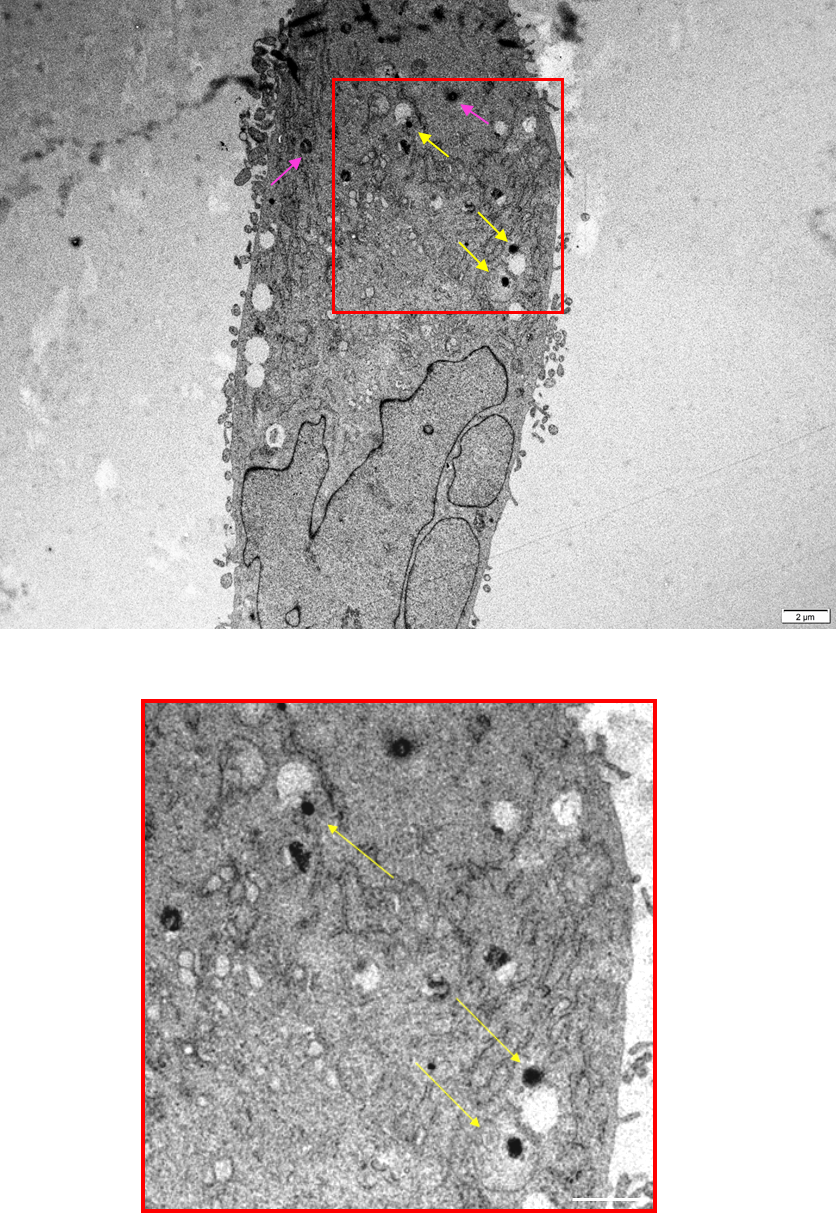


**Figure Supplementary 3**. **Electron microscopy examination of mitochondria alterations in P2 cells**. Representative images of FRDA fibroblasts (P2). FRDA cells showed mitochondrial vacuolization, and condensation/lateralization of mitochondrial membranes and lipofuscin aggregates formation (yellow arrows). FRDA cells showed the accumulation of lipofuscin-like aggregates (pink arrows). Scale bars= 2μm. Bottom panel, magnification of an area (white rectangle) of the figure in the top panel.


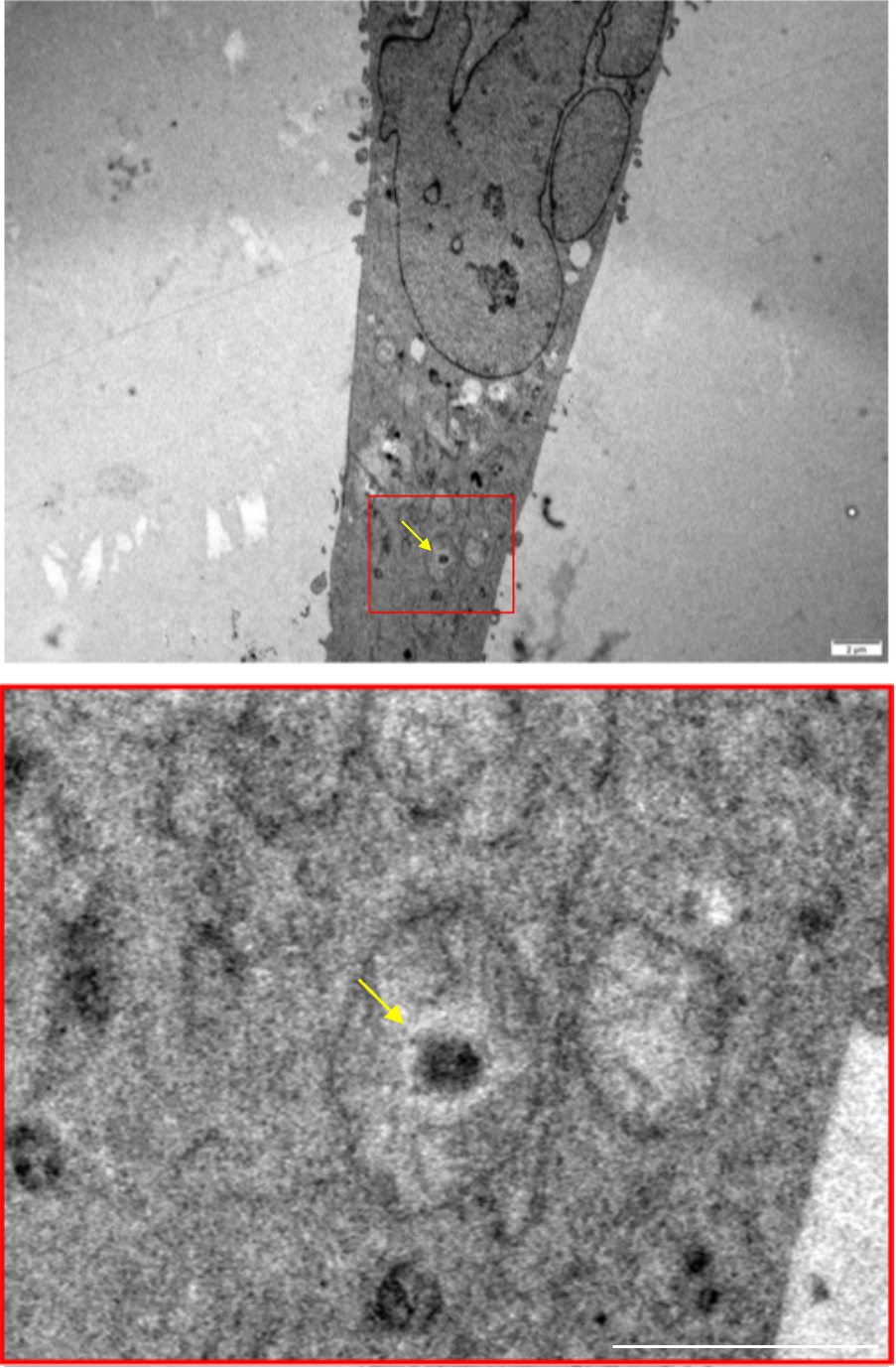


**Supplementary Figure 4. Electron microscopy examination of FRDA cells**. Representative images of P2 fibroblasts. FRDA cells showed mitochondrial vacuolization, and condensation/lateralization of mitochondrial membranes. Scale bars= 2μm. Bottom panel, magnification of an area (white rectangle) of the figure in the top panel. Lipofuscin aggregates formation inside mitochondria (yellow arrows).


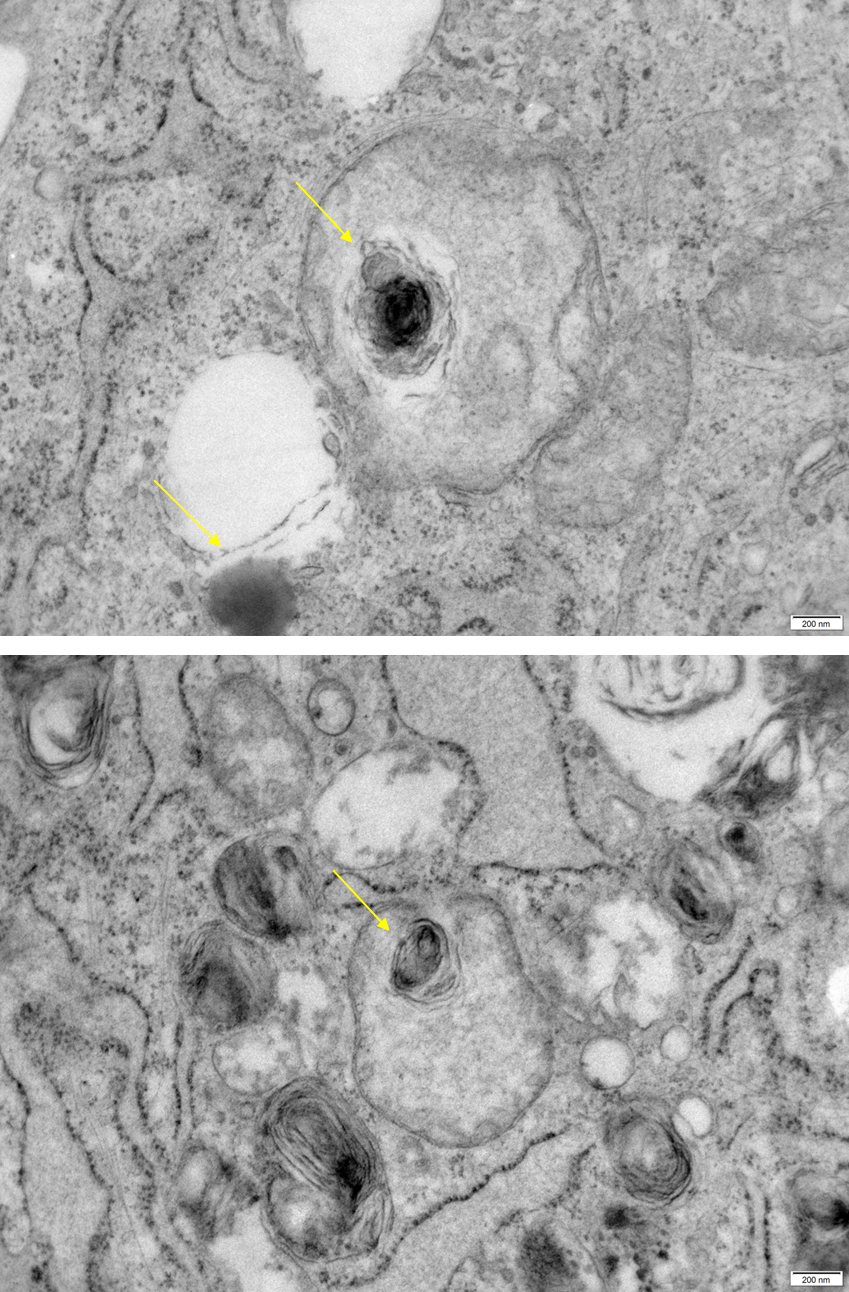


**Supplementary Figure 5. Electron microscopy examination of FRDA cells**. Representative images of P2 fibroblasts. FRDA cells showed lipofuscin aggregates formation inside mitochondria. Scale bars= 200 nm. Lipofuscin aggregates formation inside mitochondria (yellow arrows).


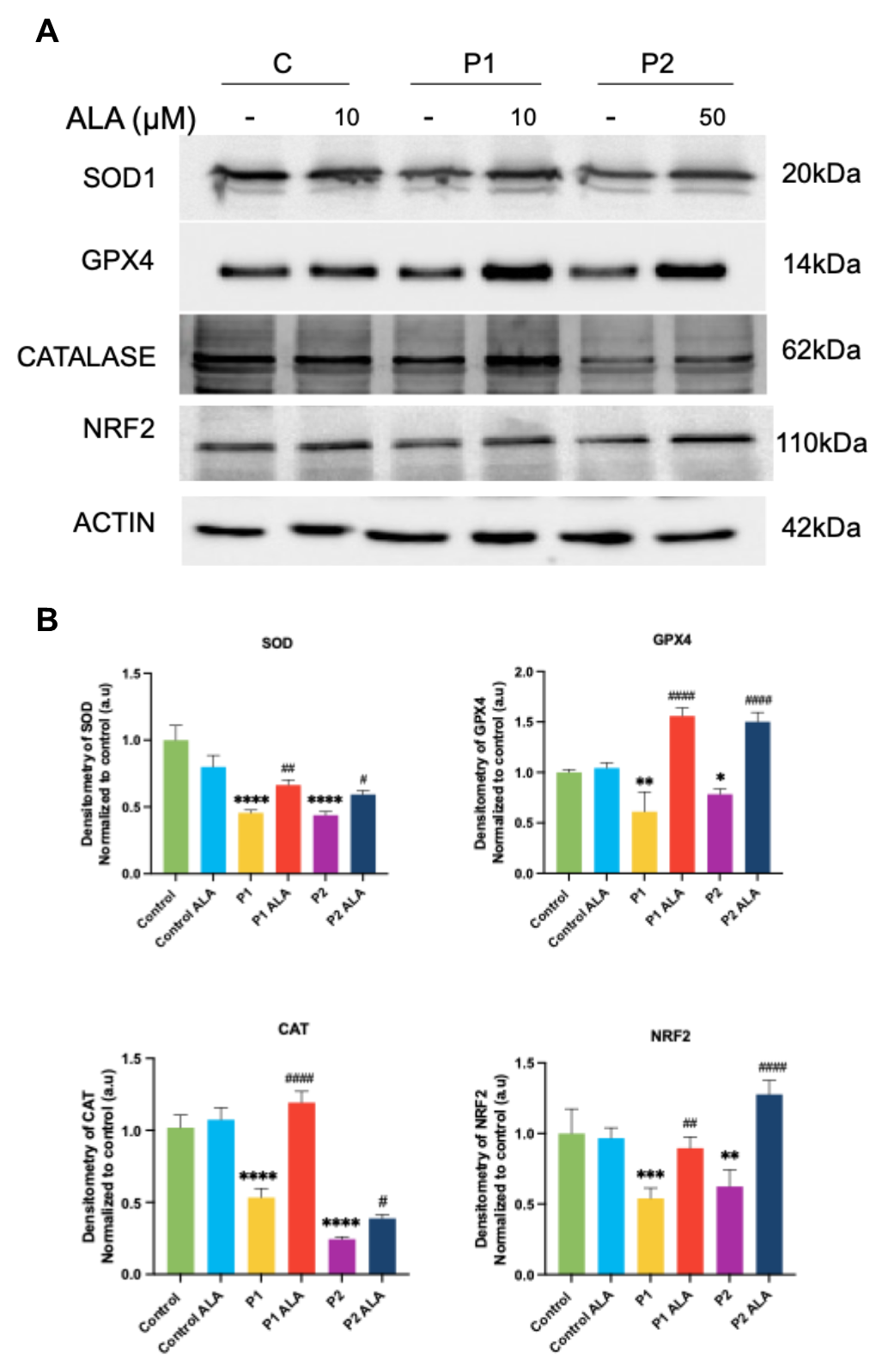


**Figure Supplementary 6. Effect of ALA supplementation on antioxidant system in control and FRDA fibroblasts. (A)** Representative images of protein expression levels involved in antioxidant mechanisms, SOD1, GPX4, CATALASE and NRF2 in control and FRDA fibroblasts (P1 and P2), untreated and treated with ALA. Actin expression levels were used as the loading control. **(B)** Densitometry of Western blot. Data is expressed as the mean ± SD of three independent experiments. Statistical significance between control and FRDA fibroblasts is represented as *p<0.05, **p<0.01, ***p<0.001, and ****p<0.0001. Statistical significance between untreated and treated fibroblasts is expressed as ^#^p<0.05, ^##^p<0.01, ^####^p<0.0001.

**Table 1. Antibodies and reagents**
